# Design of thiol–ene photoclick hydrogels using facile techniques for cell culture applications†
†Electronic supplementary information (ESI) available. See DOI: 10.1039/c4bm00187g



**DOI:** 10.1039/c4bm00187g

**Published:** 2014-09-01

**Authors:** Lisa A. Sawicki, April M. Kloxin

**Affiliations:** a Department of Chemical and Biomolecular Engineering , University of Delaware , Newark , DE 19716 , USA . Email: akloxin@udel.edu; b Department of Materials Science and Engineering , University of Delaware , Newark , DE 19716 , USA

## Abstract

Thiol–ene ‘click’ chemistries have been widely used in biomaterials applications, including drug delivery, tissue engineering, and controlled cell culture, owing to their rapid, cytocompatible, and often orthogonal reactivity. In particular, hydrogel-based biomaterials formed by photoinitiated thiol–ene reactions afford spatiotemporal control over the biochemical and biomechanical properties of the network for creating synthetic materials that mimic the extracellular matrix or enable controlled drug release. However, the use of charged peptides functionalized with cysteines, which can form disulfides prior to reaction, and vinyl monomers that require multistep syntheses and contain ester bonds, may lead to undesired inhomogeneity or degradation under cell culture conditions. Here, we designed a thiol–ene hydrogel formed by the reaction of allyloxycarbonyl-functionalized peptides and thiol-functionalized poly(ethylene glycol). Hydrogels were polymerized by free radical initiation under cytocompatible doses of long wavelength ultraviolet light in the presence of water-soluble photoinitiators (lithium acylphosphinate, LAP, and 2-hydroxy-1-[4-(2-hydroxyethoxy)phenyl]-2-methyl-1-propanone, Irgacure 2959). Mechanical properties of these hydrogels were controlled by varying the monomer concentration to mimic a range of soft tissue environments, and hydrogel stability in cell culture medium was observed over weeks. Patterns of biochemical cues were created within the hydrogels post-formation and confirmed through the incorporation of fluorescently-labeled peptides and Ellman's assay to detect free thiols. Human mesenchymal stem cells remained viable after encapsulation and subsequent photopatterning, demonstrating the utility of the monomers and hydrogels for three-dimensional cell culture. This facile approach enables the formation and characterization of hydrogels with well-defined, spatially-specific properties and expands the suite of monomers available for three-dimensional cell culture and other biological applications.

## Abstract 2

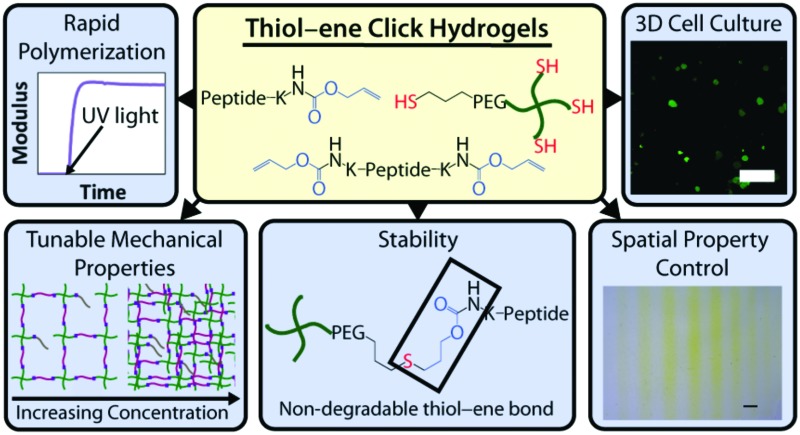
Thiol–ene photoclick hydrogels with tunable biomechanical and biochemical properties for biological applications, including controlled cell culture, regenerative medicine, and drug delivery.

## Introduction

Click chemistries for the formation and modification of biomaterials have garnered significant and growing interest for numerous applications, including drug delivery, tissue engineering, and controlled cell culture.^1,2^ A number of functional groups undergo efficient and highly selective click reactions under a variety of cytocompatible conditions, making them well suited for the manipulation of biomaterial properties in the presence of cells.^3,4^ These reactions include radically initiated thiol–ene and thiol–yne,^5,6^ thiol-Michael addition,^7,8^ spontaneous reaction of azides with strained alkynes,^9,10^ and spontaneous reaction of tetrazine with norbornene and transcyclooctene,^11,12^ which have been used to examine the effects of matrix properties on cell behavior,^6,7,9,11^ to label cells and biomolecules,^10,12^ and to form carriers for drug delivery.^13^ Amongst these, thiol–ene click chemistries have been examined broadly for the formation and modification of hydrogel-based biomaterials owing to their ease of use and the availability of thiols in many biomolecules.^14^

Hydrogels formed by thiol–ene click reactions have been constructed with a range of cytocompatible polymers and copolymers, such as poly(ethylene glycol) (PEG),^15^ hyaluronic acid,^16^ and poly(ethylene glycol)–poly(lactic acid),^17^ and modified with peptides and proteins, such as GPQG↓IWGQ,^18^ IPVS↓LRSG,^18^ and RGDS,^19^ to impart specific biological activity.^16,20^ Various vinyl functional groups have been investigated for this purpose, including norbornene,^19^ vinyl sulfone,^8^ and allyl ether.^21^ For example, the Michael-type addition of thiols on peptides with vinyl groups (‘ene's) on vinyl sulfone-modified PEG has been widely employed to design hydrogels with controlled, cell-responsive properties for use in drug delivery or tissue engineering.^8,22^ These reactions proceed *via* a step growth mechanism,^5,14^ resulting in a homogeneous network structure with robust mechanical properties for applications in cell culture and delivery.^23^

Photoinitiated thiol–ene systems are particularly attractive for hydrogel formation and modification because they allow user-directed control over the presentation of biophysical or biochemical cues in space and in time to promote specific cellular functions and toward mimicking the dynamic structure or composition of the native extracellular matrix (ECM) *in vitro*.^24,25^ Peptides modified with cysteines and polymers modified with acrylates (mixed step and chain growth mechanism) or norbornenes (step growth mechanism) have been extensively used owing to their rapid reaction under cytocompatible photopolymerization conditions.^19,26,27^ For example, Fairbanks *et al.* first demonstrated that norbornene-modified PEG reacts within minutes with cysteine-modified, enzymatically degradable crosslinking peptides in the presence of a radical initiator to form hydrogels by step growth free radical polymerization.^19^ This strategy (vinyl-modified PEG) has been used to encapsulate a number of cell types including, but not limited, to osteoblasts, chondrocytes, mesenchymal stem cells (MSCs), and smooth muscle cells.^28^ These chemistries also have been used to create new biomaterial systems, such as a hydrogel formed by the reaction of norbornene-modified hyaluronic acid with a dithiol crosslinker and modified with patterns of biochemical cues at select time points.^16^

Despite their great utility, there are a few potential concerns when using these existing thiol–ene photoclick systems. Recently, Shih and Lin observed that ester bonds present in polymers modified with various vinyl groups (*e.g.*, acrylic acid or norbornene carboxylic acid) degrade over relatively short times in water or cell culture conditions (*i.e.*, days to weeks), where the hydrolysis rate is affected by the incorporation of different charged peptide sequences.^29^ Preprogrammed degradation afforded by hydrolysis allows cell spreading within the matrix; however, it is often desirable for the rate of degradation to respond dynamically to cell secreted enzymes or an externally-applied stimulus (*e.g.*, light). Toward designing alternate systems with controlled degradation (*e.g.*, cell-secreted enzymes or light), polymer precursors modified with amine functional groups instead of hydroxyls have been utilized, introducing more water-stable amide bonds upon reaction with carboxylic acid-containing functional groups.^30,31^ Despite this increased stability, there typically is increased cost or synthetic processing associated with using these materials. Additionally, the formation of disulfide bonds between cysteine-modified charged peptides^32^ before reaction may deplete the concentration of thiols present in the reaction solution, resulting in an off-stoichiometry mixture, defects in the network structure, and slower polymerization times.^33,34^

**Fig. 1:**
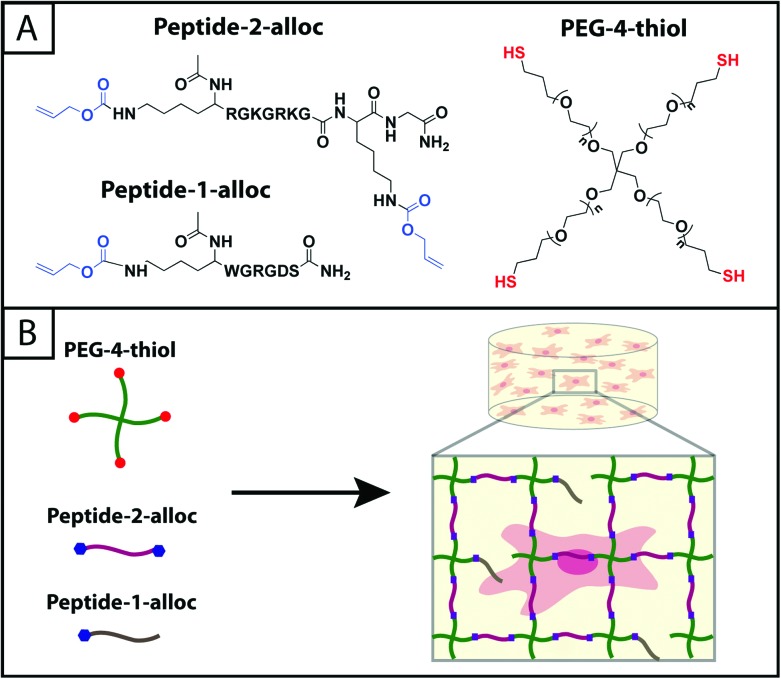
**Hydrogels formed by thiol–ene photoclick reactions for cell culture applications.** (A) Monomers functionalized with thiols or with alloc groups were synthesized for hydrogel formation using thiol–ene click chemistry: multi-armed PEG was modified with thiols (right) and peptides containing alloc-protected lysines (1 or 2) (left). Upon the application of light, these functional groups react by a step growth mechanism, where an initiating species generates a thiyl radical that attacks the pendant ‘ene’ and forms a stable covalent bond between the monomers in solution.^19^ (B) This material system is promising for cell encapsulation and three-dimensional cell culture, where the thiol-modified PEG is crosslinked with alloc-containing peptides in the presence of cells allowing their encapsulation for *in vitro* studies. Capitalizing on the spatial control enabled by the thiol–ene photoclick reaction, pendant peptides (containing one alloc) can be added within the network during or after gel formation to promote cell–matrix interactions.

Herein, an approach to rapid thiol–ene photoclick polymerization between a vinyl-modified peptide and thiol-modified PEG is presented (Fig. 1). A multiarm PEG thiol is used as the ‘backbone’ of the hydrogel structure with thiols on each arm connected by ether bonds. The PEG backbone is not charged, limiting potential disulfide formation,^35^ and ether bonds neighboring thiol functional groups provide a water-stable base for the introduction of enzymatically degradable peptide sequences for cell-dictated degradation. The alloxycarbonyl (alloc) group, which is used to protect the amines of amino acids (*e.g.*, lysine) during peptide synthesis, is incorporated within pendant (single)^15,36,37^ and crosslink (double) peptide sequences to provide vinyls for reaction with the PEG thiol backbone. The use of lithium acylphosphinate (LAP) as a photoinitiator, which has increased rates of initiation and polymerization relative to other water-soluble photoinitiators,^38^ allows the rapid reaction of the alloc-modified peptides with the multiarm PEG thiol to form hydrogels under cytocompatible doses of long wavelength ultraviolet (UV) light (10 mW cm^–2^, 365 nm).^39^ Further, these monomers may be purchased commercially or synthesized with relatively simple techniques presented here, making the system accessible to researchers in a variety of fields. In this article, the polymerization, mechanical properties, stability, cytocompatibility, and spatial patterning of these robust thiol–ene photoclick hydrogels are characterized to define and demonstrate their potential for use as three-dimensional (3D) mimics of the ECM, particularly for the evaluation of cell–matrix interactions. In addition to the application of these materials in controlled cell culture models, we believe this approach may be useful for the *in situ* modification of assembling peptides (*e.g.*, adding functionalities to supramolecular structures to allow electrical conduction, enhance imaging, or promote specific biological interactions)^40,41^ and even in membrane applications (*e.g.*, forming stable, charged PEG-based membranes for batteries).^42^

## Materials and methods

### Synthesis of PEG-thiol macromer

Poly(ethylene glycol)-tetrathiol (PEG4SH) is commercially available (JenKem Technology USA, Creative PEGWorks) or can be synthesized as was done here using a modified version of published protocols.^43^ Briefly, four-arm PEG (*M*_n_ ∼ 20 000 g mol^–1^, 10 g) (JenKem USA) was dissolved in anhydrous tetrahydrofuran (THF, 70 mL) (Fisher Scientific) and purged with argon, and argon-purged sodium hydroxide (NaH, 4× molar excess with respect to –OH groups) (Sigma Aldrich) suspended in THF was transferred *via* cannula under argon to the dissolved PEG. Allyl bromide (3× molar excess with respect to –OH groups) (Acros Organics) dissolved in 30 mL of THF subsequently was added. The PEG-allyl solution was refluxed overnight at 40 °C under argon and precipitated in ice cold ethyl ether to generate allyl ether-modified PEG (PEG4AE). The PEG4AE was dissolved in dichloromethane (40 mL) (Fisher Scientific) with a photoinitiator (2,2-dimethoxy-1,2-diphenylethan-1-one, I651, 0.5% w/w) (Acros Organics) and trace trifluoroacetic acid (TFA, ∼100 μL) (Acros Organics) and purged with argon. Thioacetic acid (2× molar excess with respect to allyl) (Acros Organics) was added, and the solution was purged with argon and subsequently exposed to UV light (365 nm at 10–15 mW cm^–2^ for 45 minutes) to yield PEG-thioacetate (PEG4TA) after precipitation in ice cold diethyl ether. Last, PEG4TA was dissolved in 60–70 mL of water and purged with argon. An equal volume of 1 M sodium hydroxide (Fisher Scientific) purged with argon was added to the PEG4TA (0.5 M final concentration) to generate the thiol end groups on the final PEG4SH product. The reaction immediately was neutralized with hydrochloric acid (final pH 1–2) (Fisher Scientific) and PEG4SH extracted with chloroform and trace TFA (to prevent disulfide formation) and precipitated in ice cold diethyl ether. To wash and collect all intermediates and the final product after precipitation, samples were centrifuged at 0 °C for 20 minutes at 4400 rpm for a total of 3 washes and dessicated under vacuum at room temperature overnight. All intermediates and the final product were characterized with proton nuclear magnetic resonance (^1^H NMR) in DMSO: PEG4AE 5.1–5.2 (m, 1H) 5.2–5.3 (m, 1H) 5.8–5.9 (m, 1H); PEG4TA 2.3 (s, 3H); PEG4SH 2.3 (m, 1H) for a single arm of the tetrafunctional monomer (ESI Fig. S1†).

### Synthesis of alloc-functionalized peptides

The pendant cell adhesion sequence **K(alloc)**GWGRGDS (RGDS), a ubiquitous sequence found in many ECM proteins including fibronectin and vitronectin,^44^ was synthesized to promote cell adhesion (amino acid(s) with reactive functional groups in bold). Non-degradable, water-soluble crosslinking sequences were synthesized: **K(alloc)**RGKGRKG**K(alloc)**G^37^ (RGKGRK2alloc) (primary sequence used in hydrogel development) and **K(alloc)**GKGWGKG**K(alloc)**G (GKGWGKG2alloc) and **C**GKGWGKG**C**G (GKGWGKG2SH) (sequences with reduced charge and including tryptophan for easily assessing their concentration). Additionally, an enzymatically degradable, water-soluble crosslinking sequence K**K(alloc)**GGPQG↓IWGQG**K(alloc)**K (GPQGIWGQ2alloc) (broadly degradable by matrix metalloproteinases (MMP)-1, 2, 3, 8 and 9)^18^ was synthesized to promote cell viability and allow spreading in longer cell culture and photopatterning experiments. Each was synthesized by standard solid phase peptide synthesis (SPPS) techniques using Fmoc chemistry on MBHA rink amide resin (0.59 mmol g^–1^; 0.25 mmol scale) (Novabiochem) with a peptide synthesizer (Protein Technologies PS3). Fmoc-protected amino acids, including the commercially-available alloc-protected lysine, and *o*-(benzotriazol-1-yl)-*N*,*N*,*N*′,*N*′-tetramethyluronium hexafluorophosphate (HBTU) (4× excess) (Chem-Impex International) were loaded into cartridges and coupled on resin. Fmoc deprotection was carried out using 20% piperidine (Sigma Aldrich) in *N*,*N*-dimethylformamide (DMF) (Fisher Scientific) prior to each amino acid coupling in 0.4 M methylmorpholine in DMF. Peptide products were cleaved in 95% v/v trifluoroacetic acid (TFA), 2.5% v/v triisopropylsilane (TIPS) (Acros Organics), and 2.5% v/v water with 5% w/v dithiothreitol (DTT) (Research Products International Corporation) to prevent disulfide formation and 2.5% w/v phenol (Sigma Aldrich) to protect tryptophan (W). After cleavage from the resin, peptides were precipitated in ice cold diethyl ether, centrifuged at 3000 rpm and 4 °C for 5 minutes for a total of three washes and dessicated under vacuum overnight at room temperature. Dry raw peptide product was purified by high-performance liquid chromatography (HPLC) and analyzed by matrix-assisted laser desorption/ionization (MALDI, crystallized with α-cyano-4-hydroxycinnamic acid, Acros Organics) or electrospray ionization (ESI) mass spectrometry to confirm synthesis of each desired peptide (ESI Fig. S2†).

A fluorescently-labeled pendant peptide, Alexa Fluor 488-AhxWGRGDS**K(alloc)**G (AF488RGDS), also was designed for photopatterning experiments using published protocols.^37^ After Fmoc deprotection of Ahx on the N′-terminus of the peptide, 1 mg Alexa Fluor® 488 Carboxylic Acid, 2,3,5,6-Tetrafluorophenyl Ester, 5-isomer (Invitrogen) was stirred with 0.25 mmol peptide on resin in 4 mL DMF and 50 μL *N*,*N*′-diisopropylethylamine (DIPEA) (Chem-Impex International) overnight. The peptide was cleaved from resin, precipitated, and analyzed by HPLC and ESI mass spectrometry (ESI Fig. S2†).

### Synthesis of LAP initiator

The LAP initiator was synthesized using previously-described methods.^38^ Briefly, 2,4,8-trimethylbenzoyl chloride (1.6 g, 0.009 mol) (Sigma Aldrich) was added to dimethyl phenylphosphonite (1.5 g, 0.009 mol) (Acros Organics) and reacted overnight at room temperature under argon. Lithium bromide (4× molar excess) (Sigma Aldrich) in 2-butanone (Sigma Aldrich) was added to the reaction solution and heated to 50 °C for 10 minutes. The white precipitate was filtered and rinsed 3 times with 2-butanone, and the final powder product dried and analyzed by ^1^H NMR, matching literature (ESI Fig. S3†).^38^

### Hydrogel formation

All monomers and initiators were prepared in Dulbecco's phosphate buffered saline (PBS) (Life Technologies) immediately before polymerization. For the various experiments described below, solutions of PEG4SH, RGKGRK2alloc (unless noted otherwise), and RGDS (7.5, 10, 12.5 wt% with respect to PEG, 2 mM RGDS) were prepared at stoichiometric ratios of thiol functional groups to alloc functional groups (1 : 1 SH : alloc) and containing a photoinitiator, either LAP (1.1 and 2.2 mM) or Irgacure 2959 (I2959) (2.2 mM). Hydrogels were formed upon irradiation of the monomer-initiator solution with cytocompatible doses of long wavelength UV light (365 nm at 10 mW cm^–2^, International Light IL1400A Radiometer/Photometer) in the specific geometries described below.

### Rheometry

Hydrogels were formed *in situ* on a photorheometer (TA AR-G2 with UV light attachment, Exfo Omnicure Series 2000 light source, 365 nm filter, SilverLine UV Radiometer M007-153) to estimate the polymerization times for different initiator types and monomer concentrations. I2959 (2.2 mM) or LAP (1.1 or 2.2 mM) photoinitiators were added to 10 wt% PEG monomer solutions containing stoichiometrically balanced amounts (1 : 1 SH : alloc) of RGKGRK2alloc to compare the effects of initiator type on polymerization time (*n* = 3). PEG monomer solutions (7.5, 10, and 12.5 wt%) containing stoichiometrically balanced amounts of RGKGRK2alloc and RGDS (2 mM) were mixed with 2.2 mM LAP to compare the effects of monomer concentration on polymerization time (*n* = 6). Finally, PEG4SH or PEG4AE monomer solutions (10 wt%) containing stoichiometrically balanced amounts of alloc (RGKGRK2alloc, GKGWGKG2alloc, GPQGIWGQ2alloc) or thiol-modified crosslinkers (PEG2SH, GKGWGKG2SH) were mixed with 2.2 mM LAP to compare the effects of crosslinker and functional group chemistry on polymerization time (*n* = 3). These solutions were placed between parallel plates (8 mm diameter, 200 μm gap) and UV light (365 nm at 10 mW cm^–2^) applied 1 minute after starting rheometric measurements. Storage (*G*′) and loss moduli (*G*′′) were recorded over time at 2% applied strain and 6 rad s^–1^ frequency. From the data, an approximate time for complete gelation was defined to be when the percent change in modulus between consecutive data points was less than 0.1%.

For swollen modulus experiments, 7.5, 10, and 12.5 wt% hydrogels were polymerized within a 1 mm thick mold (2 microscope slides treated with Rain-X separated by a 1 mm rubber gasket). After polymerization, discs (8 mm diameter) were punched from the gel slab and swollen overnight in PBS. Strain sweeps (1 rad s^–1^ frequency, 1–100% strain) and frequency sweeps (1–100 rad s^–1^ frequency, 5% strain) were conducted on swollen gels to determine the linear viscoelastic regime for the material. The swollen gels were then placed between parallel plates on the rheometer and *G*′ and *G*′′ were measured at 5% strain and 5 rad s^–1^ frequency (within the linear viscoelastic regime) (*n* = 6).

### Hydrogel swelling

Experiments to determine volumetric swelling ratios (*Q*) were performed on 7.5, 10, and 12.5 wt% hydrogels. Discs (8 mm diameter) were punched from gels polymerized between glass slides separated by a 1 mm thick gasket, ensuring sufficient mass for measuring dry weight, and swollen overnight in PBS. After recording swollen mass (*M*_s_), the gels were lyophilized and the dry masses were measured (*M*_d_) (*n* = 6). Volumetric swelling ratio was calculated by the relationships:
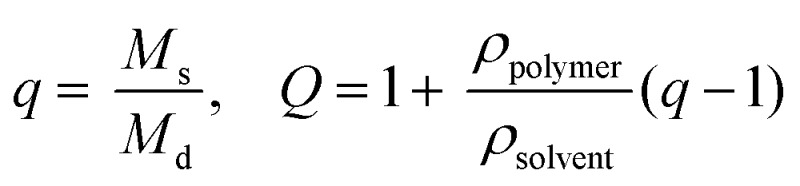
where *q* is the mass swelling ratio, *ρ*_polymer_ = 1.07 g mL^–1^ ^45^ for PEG, and *ρ*_solvent_ = 1.00 g mL^–1^ for PBS.

Experiments to determine gel stability after polymerization were performed on gels incubated in PBS and cell culture medium at 37 °C over a 3 week time course. Gels (10 wt%) were polymerized for 5 minutes in 5 mm diameter molds (1 mL syringes with tips cut off) under sterile conditions and placed in sterile PBS and cell culture medium. *M*_s_ and *M*_d_ were recorded for the gels after 1, 7, 14, and 21 days (*n* = 6). Values for the volumetric swelling ratio (*Q*) were calculated as described above.

### Detection of unreacted thiols

To initially quantify the photoaddition of biochemical cues, hydrogels (10 wt% with respect to PEG) were polymerized (1 or 5 minutes) between glass slides separated by a 0.254 mm thick gasket (McMaster-Carr) and off-stoichiometry such that approximately 2 mM free thiol remained in the unswollen gel after polymerization. Discs (5 mm diameter) were punched from these gels for further treatment and analysis. Half of the gel discs were swollen in PBS containing LAP initiator (2.2 mM) and excess pendant peptide (20 mg mL^–1^, K(alloc)GWGRGDS) and incubated at room temperature for 1 hour. After 1 hour, these gels were exposed to UV light for 1 or 5 minutes to initiate the photoaddition of the RGDS. The other half of the gels remained in PBS as a control. Free thiol concentrations in the gels were quantitatively detected by Ellman's assay as described below.

Briefly, the swollen volume of the gels was predicted using the measured *Q* value (estimated at 19.3 μL). Ellman's reaction buffer (20.7 μL) containing 0.1 M sodium phosphate (Sigma Aldrich) and 1 mM ethylenediaminetetraacetic acid (Sigma Aldrich) at pH 7.5–8 was added to the gels for a total volume of 40 μL. Ellman's reagent (7.2 μL, 4 mg in 1 mL reaction buffer) (Fisher Scientific) was diluted in 360 μL of reaction buffer and added to each well containing a gel. Gels were incubated in the reagent for 1 hour and 30 minutes, the estimated time for the diffusion of the yellow NTB^2–^ dianion out of the gel so that the supernatant and gel colors match (by visual inspection). Finally, a calibration curve of l-cysteine hydrochloride monohydrate (Sigma Aldrich) (0–2 mM) was made to calculate the concentration of thiols detected in each gel. Absorbance of each condition was measured at 405 nM (Biotek Synergy H4 automated plate reader).

To determine the free thiol concentration in conditions for photopatterning in the presence of encapsulated cells, 10 wt% gels were polymerized in syringe tips (20 μL) such that approximately 2 mM free thiol remained in the unswollen gel after polymerization. Gels polymerized for 1 and 5 minutes were placed immediately in PBS as a control (*n* = 3). Additional gels polymerized for 1 minute immediately were placed in solutions of PBS containing 3 mg mL^–1^ RGDS and 2.2 mM LAP and incubated at 37 °C for 30 minutes (*n* = 3) or 1 hour 30 minutes (*n* = 3). After incubation, these gels were exposed to a second dose of UV light for 1 minute to attach the biochemical cue (RGDS) to remaining free thiols. Free thiol concentrations in the gels were quantitatively detected by Ellman's assay as described above, accounting for larger gel size (swollen volume = 84.8 μL; add 15.2 μL of PBS to gel in well plate for 100 μL total volume; add 18 μL Ellman's reagent in 900 μL Ellman's buffer to each well).

### Spatially-specific photopatterning of biochemical cues

Hydrogels (10 wt%) were polymerized between glass slides spaced by a 0.254 mm gasket and off-stoichiometry to have a final free thiol concentration of 2 mM within the as prepared gel (prior to equilibrium swelling). The hydrogel was left on one of the glass slides for subsequent treatments and rinsed with PBS for 1 hour. Rinsed gels were placed in solution containing pendant peptides (AF488RGDS or RGDS) mixed with 2.2 mM LAP initiator for 1 hour and 30 minutes to allow diffusion of the peptides and initiator into the gel network prior to subsequent patterning. Photomasks with lines of increasing thickness (0.2–1 mm width) or square patterns (0.4 mm edge) purchased from Advanced Reproductions Corporation were placed ink-side down on top of the samples and exposed to collimated UV light (Inpro Technologies collimating adaptor, Exfo Omnicure Series 2000 light source) for 1 minute (365 nm at 10 mW cm^–2^). Gels were rinsed 3× for 40 minutes each with PBS to remove excess pendant peptide after photoaddition. Samples containing the patterned AF488RGDS were imaged with a confocal microscope (Zeiss 510 NLO). Ellman's reagent was applied to the gels containing RGDS and imaged immediately on a stereomicroscope (Zeiss Stemi 2000-C).

### Culture and encapsulation of human mesenchymal stem cells

Human mesenchymal stem cells (hMSCs) isolated from human bone marrow (Lonza)^46^ were cultured on tissue-culture treated polystyrene in cell culture medium^46^ and harvested at ∼70–80% confluency (Passage 2, 3) for experiments. For evaluating the effects of light, cells were trypsinized from culture plates, counted (hemacytometer), centrifuged (5 minutes, 1000 rpm), and plated at a density of 20 000 cells cm^–2^ in 96-well plates. For cell encapsulation and photopatterning experiments, cells were trypsinized from culture plates, counted (hemocytometer), centrifuged (5 minutes, 1000 rpm), and resuspended at desired densities in monomer solution (10 wt%) with and without RGDS. The mixtures of cells in monomer solution were polymerized in syringe molds at cytocompatible wavelengths and doses of UV light (365 nm at 10 mW cm^–2^), encapsulating cells within the hydrogel matrix.

### Metabolic activity of hMSCs in photopatterned and non-patterned hydrogels

Cells were suspended in monomer solution (10 wt%, 3000 cells μL^–1^) containing 2 mM RGDS and polymerized in syringe tip molds (20 μL) for 1 and 5 minutes (*n* = 6, non-patterned). Immediately after polymerization, gels were placed in cell culture medium to rinse out unreacted monomer and photoinitiator (30 minutes). After rinsing, the medium was replaced with fresh medium and gels were incubated at 37 °C for subsequent analysis. For photopatterned gels, cells were suspended in monomer solution (10 wt%, 3000 cells μL^–1^) without RGDS and polymerized for 1 minute such that 2 mM free thiols remained in the unswollen gel for subsequent modification. After polymerization, the gels were incubated in PBS containing 3 mg mL^–1^ RGDS and 2.2 mM LAP for 30 minutes or 1 hour 30 minutes at 37 °C before exposure to a second dose of UV light (1 minute) to covalently link RGDS within the network (*n* = 6). Patterned gels were immediately placed in cell culture medium (30 minutes) to rinse out excess monomer and photoinitiator. At 1 and 3 days post-encapsulation (D1 and D3), metabolic activity was assessed by CellTiter 96 (Promega) (*n* = 3 each condition, each time point).

To assess the effect of light alone on cell function, plated cells (20 000 cells cm^–2^) were exposed to UV light (1 min of 365 nm at 10 mW cm^–2^). Metabolic activity was assessed by CellTiter 96 at D1 and D3 compared to control (no light) (*n* = 3 each condition, each time point).

### Viability of hMSCs in photopatterned and non-patterned hydrogels

To initially study the viability of cells encapsulated in hydrogels, 3000 cells μL^–1^ were encapsulated in non-degradable gels (10 wt%, 2 mM RGDS before swelling) polymerized for 1 and 5 minutes. Additional studies were performed to determine the effect of cell density on viability post-encapsulation, with cells encapsulated in non-degradable gels (10 wt%, 2 mM RGDS before swelling) at 3000 and 30 000 cells μL^–1^. Viability was quantified at 3 days post-encapsulation with a LIVE/DEAD® Viability/Cytotoxicity Kit for mammalian cells (Invitrogen), and gels were imaged with a confocal microscope (Zeiss 510 NLO).

To study the viability of cells in photopatterned hydrogels over longer times in culture, cells were encapsulated in gels (10 wt%, 20 μL, 3000 cells μL^–1^) crosslinked with the degradable (GPQGIWGQ2alloc) peptide sequence such that 2 mM free thiol remained in unswollen gels post-polymerization (1 minute). Gels were placed in PBS containing 3 mg mL^–1^ RGDS and 2.2 mM LAP for 1 hour and exposed to a second dose of UV light (1 minute) to allow attachment of RGDS to the network. Viability was assessed 6 days after encapsulation with the LIVE/DEAD® Viability/Cytotoxicity Kit for mammalian cells, providing time for hMSCs to partially degrade and attach to the hydrogel matrix.

## Results and discussion

Click chemistries for hydrogel formation are of interest in many biomaterials applications. Their efficient reactions under mild conditions enable hydrogel formation and modification in the presence of proteins and cells,^3,28^ which is especially useful for designing materials that mimic native tissue environments *in vitro* for cell culture. Light-mediated thiol–ene click reactions in particular are of great utility for control over the presentation of biomechanical and biochemical cues in space and time within these systems. Here, we describe a new approach to utilizing thiol–ene chemistry for hydrogel formation and spatially-specific patterning in cell culture applications with alloc-functionalized peptides and thiol-terminated PEG. This strategy enables rapid and consistent polymerization of hydrogels controlled by the application of light, the formation of a stable bioinert base matrix, and the spatial presentation of biochemical cues within the hydrogel network.

### Initiator selection allows rapid polymerization under cytocompatible conditions

Thiol–ene reactions for biomaterial applications can occur spontaneously in aqueous solutions in the presence of a base catalyst or upon the introduction of free radicals, depending on vinyl group selection.^14,47^ For example, base-catalyzed polymerization of hydrogels in the presence of cells by Michael-type addition reactions between thiols and vinyl sulfones or maleimides has been used to understand cell behavior, invasion, and differentiation in synthetic mimics of the ECM.^7,22^ Additionally, for control over when and where the reaction takes place, polymerizations of hydrogels by a photoinitiated, free radical step growth reaction between thiols and vinyls (*e.g.*, norbornene) have been used with cytocompatible doses of UV or visible light depending on initiator selection (*e.g.*, Irgacure 2959,^39^ lithium acylphosphinate,^38^ or Eosin Y^30^). While the spatiotemporal control afforded by photopolymerization is quite useful, minimizing exposure to light, particularly wavelengths in the UV, is crucial for polymerizations done in the presence of cells.^39,48^ Light-mediated reaction conditions that are cytocompatible and rapid for the polymerization of monomers in aqueous solutions often are limited and are needed to reduce the exposure time of cells and proteins to light and reactive components (particularly free radicals). Toward addressing this, we aimed to establish conditions for the photopolymerization of monomers functionalized with thiols and allocs to expand the suite of reactions for cell encapsulation.

Previously, the general reaction of allyl- and thiol-functionalized monomers for hydrogel formation was considered too slow for gel formation in the presence of cells, which may be due to a rate-limiting chain transfer step,^49^ and has been described with limited use in cell culture applications for the modification of synthetic hydrogel matrices with pendant alloc-modified peptide tethers.^15,37^ Here, we examined water-soluble initiator and monomer compositions to identify cytocompatible conditions for alloc-based hydrogel formation. Hydrogels were polymerized *in situ* on a rheometer to monitor polymerization times of gels formed with different water-soluble photoinitiators (LAP and I2959) and initial monomer concentrations (7.5, 10, 12.5 wt% with respect to PEG). Two initiator concentrations were selected (1.1 and 2.2 mM) to match concentrations that have been used to polymerize other types of hydrogels in the presence of cells,^39^ as cell viability previously has been observed to be sensitive to the concentration of LAP owing to robust free radical generation with irradiation at 365 nm.^38^

**Fig. 2:**
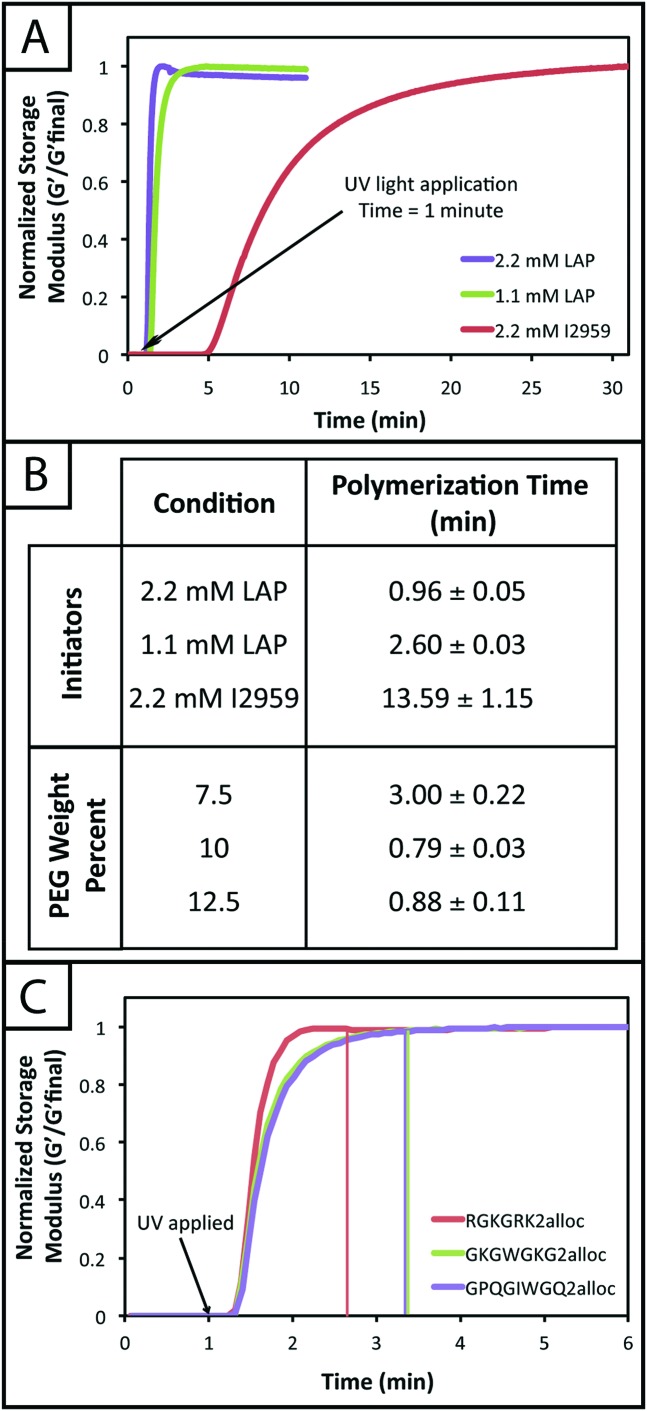
***In situ* polymerization of PEG hydrogels with different photoinitiators.** (A) Hydrogels (10 wt% with respect to PEG) were polymerized *in situ* on a rheometer to monitor gel formation over time using various initiator conditions. After 1 minute on the rheometer, UV light (10 mW cm^–2^, 365 nm) was applied to samples. The storage moduli of gels polymerized using 1.1 and 2.2 mM LAP initiator begin to increase within 30 seconds after light application and finish forming in approximately 1–3 minutes (modulus levels off). Gels polymerized using 2.2 mM I2959 begin to form 4 minutes after UV light application and reach complete formation in approximately 13–14 minutes of exposure. The rapid polymerizations observed for the LAP initiator are relevant for cell culture applications. Representative data for each condition is shown here. (B) Complete polymerization, defined here as the point where the change in modulus between consecutive data points is less than 0.1%, was determined for various initiator and monomer concentrations. As shown in (A), the LAP initiator exhibits the most rapid polymerization, with times of 0.96 ± 0.05 and 2.60 ± 0.03 minutes for 2.2 and 1.1 mM LAP, respectively. Complete polymerization for I2959, at the highest concentration compared to LAP (2.2 mM), occurs in 13.59 ± 1.15 minutes. Using 2.2 mM LAP, hydrogels from various initial monomer concentrations (7.5, 10, and 12.5 wt% with respect to PEG) were polymerized. The 7.5 wt% condition exhibited the slowest polymerization rate of 3.00 ± 0.22 minutes due to fewer functional groups that are available to react. The 10 and 12.5 wt% gels polymerized in 0.79 ± 0.03 and 0.88 ± 0.11 minutes as the number of functional groups in solution is higher at the start of polymerization. (C) Hydrogels were polymerized with different alloc-modified peptides to evaluate any effect of peptide chemistry on polymerization rate. The less charged GKGWGKG2alloc and GPQGIWGQ2alloc peptides take 40 seconds longer to polymerize than the charged RGKGRK2alloc peptide, although this is not as significant as the effects of weight percent and the type and concentration of initiator on polymerization time.

The rheological data collected by *in situ* polymerization of hydrogels demonstrates the efficiency of the LAP initiator for the radical reaction of thiol with vinyl functional groups. The slope of the moduli over time for the 1.1 and 2.2 mM LAP conditions becomes approximately 0 after complete gelation, whereas the I2959 continues to slowly increase (slope = 1.5 to 5 Pa s^–1^) (Fig. 2a) indicating a less rapid reaction. While presentation of hydrogel moduli (*y*-axis) on a log scale is typical, we have chosen to present moduli on an absolute (normalized) scale to demonstrate the efficiency of the LAP initiator in achieving complete gelation when compared directly to I2959. Further, the polymerization times of the gels formed using 1.1 and 2.2 mM LAP were determined to be approximately 5 and 15 times faster than those using I2959 as the initiator (2.60 ± 0.03 and 0.96 ± 0.05 min, respectively, *vs.* 13.59 ± 1.15 min) (Fig. 2b). This order of magnitude difference in polymerization time is comparable to differences observed between LAP and I2959 in the polymerization of other functional groups, such as the chain growth polymerization of PEG-diacrylate with LAP (10 times faster than with I2959),^38^ and arises from the increased absorbance of and radical generation by LAP relative to I2959 at long wavelengths of UV light (365 nm). Moving forward, we focused on the 2.2 mM LAP polymerization condition, which provided the most rapid gel formation. However, the 1.1 mM LAP condition may be attractive for investigations in the future for specific cell culture applications as higher initiator concentrations can result in lower cell viability.^39^

In addition to comparing the effect of different initiating conditions on polymerization rates, the concentration of monomers initially present also must be considered. The availability of terminal functional groups for reaction influences the time to complete gelation, especially at low concentrations where the distance between functional groups is greater and, after reaction of one end group, can decrease the probability of reaction with a functional group on a different monomer.^50^ We observe that the lowest initial monomer concentration (7.5 wt%) corresponds to the longest polymerization time (3.00 ± 0.22 min) while the higher concentrations (10, 12.5 wt%) polymerize in shorter time periods (0.79 ± 0.03, 0.88 ± 0.11 min) (Fig. 2b). The polymerization time for the 7.5 wt% gels is statistically different from the 10 and 12.5 wt% gels (*p* < 0.05); however, the 10 and 12.5 wt% gels are not (*p* > 0.05). The more rapid polymerization times of the higher concentration conditions may be attributed to the increased concentration of functional groups.

Finally, we investigated the polymerization of several different alloc-modified peptides (RGKGRK2alloc, GKGWGKG2alloc, GPQGIWGQ2alloc) with PEG4SH to understand if there may be any effects of peptide sequence on polymerization time (Fig. 2c). We observed the most rapid polymerization with the highly charged RGKGRK2alloc crosslinking peptide followed by the less charged GPQGIWGQ2alloc and GKGWGKG2alloc peptides (40 seconds slower), indicating that charge may play a role in the polymerization of the system and should be considered when designing and utilizing different peptide sequences. All peptides led to complete gelation within 2.5 minutes after UV light was applied and, consequently, are promising and appropriate for cell encapsulation, as discussed further below. We briefly compared to the polymerization of PEG4AE with different thiol-containing crosslinkers (PEG2SH, GKGWGKG2SH) to examine the effect of monomer chemistry on the polymerization rate (ESI Fig. S4†). The polymerization of this ‘inverse’ system was consistently slower than the alloc system, which may be related to the reactivity of the allyl and thiol groups being affected by neighboring substituents (*i.e.*, oxycarbonyl [alloc] *vs.* ether [AE]^51^ or neighboring amino acids^52^). Further, in our hands, we observe variability in the final moduli and polymerization times for PEG4AE and peptide2SH gels, which we speculate is partially due to the propensity for disulfide formation between thiols on these charged peptides.^32^ Presentation of thiols from PEG, as demonstrated with the peptide2alloc system, allows consistent formation of hydrogels under cytocompatible conditions.

### Hydrogel mechanical properties tuned to mimic soft tissue environments

**Fig. 3:**
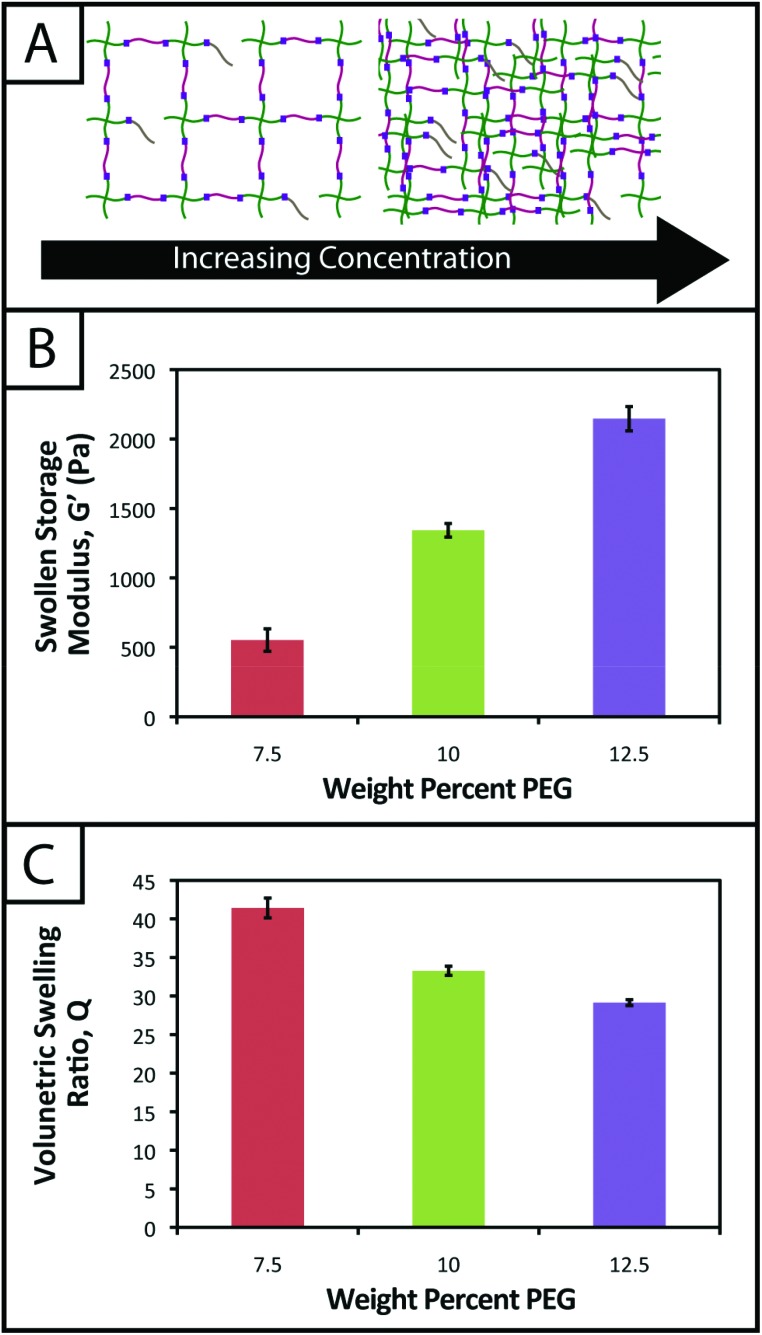
**Hydrogel mechanical properties tuned to mimic soft tissue environments.** (A) To adjust the mechanical properties of hydrogels, monomer concentration in solution prior to polymerization may be increased or decreased to increase or decrease crosslink density and thus modulus, respectively. (B) Hydrogels from different initial monomer concentrations (7.5, 10, and 12.5 wt% with respect to PEG) were polymerized and swollen in PBS to demonstrate tunable hydrogel mechanical properties. The lowest average swollen storage modulus was observed for the 7.5 wt% gels (553 ± 81 Pa), and increased with higher monomer concentrations (1343 ± 49 Pa and 2147 ± 87 Pa). (C) Another important property to consider in the design of controlled hydrogel mimics of the ECM is the volumetric swelling ratio. In accordance with modulus measurements, for the gels polymerized with an initial monomer concentration of 12.5 wt% (with respect to PEG), the lowest volumetric swelling ratio is observed (29.2 ± 0.4), and the volumetric swelling ratio increases for lower concentrations (7.5 and 10 wt% with respect to PEG) (*Q* = 41.4 ± 1.3 and 33.3 ± 0.6).

One common approach used to control or tune the initial mechanical properties of hydrogels is varying the monomer concentration.^53^ Controlling the hydrogel mechanical properties, as measured by modulus, can be critical in cell culture and regenerative medicine applications, where the elasticity, or “stiffness”, of the microenvironment that surrounds a cell has been shown to affect cell function and fate.^54,55^ These properties also must be consistent from gel-to-gel for a well-defined, controlled material system. Here, we aimed to establish hydrogel compositions with a range of equilibrium-swollen moduli that mimic different soft tissues. Toward this, we measured the swollen storage moduli (*G*′) and volumetric swelling ratios (*Q*) of hydrogels formed from different initial monomer concentrations (7.5, 10, and 12.5 wt% with respect to PEG) (Fig. 3).

We demonstrate for our material system that, by increasing the concentration of monomer in the gel-forming solution, we can increase the modulus (Fig. 3b; 7.5 wt%, *G*′ = 553 ± 81 Pa; 10 wt%, *G*′ = 1343 ± 49 Pa; 12.5 wt%, *G*′ = 2147 ± 87 Pa), creating gels with a range of elasticity comparable to native soft tissues (around the range of neural tissues to muscle, *E* ∼ 1 to 10 kPa, where *E* ≈ 3 G).^55^ Assuming that the theory of rubber elasticity holds for these swollen gels, the behavior can be attributed to an increase in crosslink density (*ρ*_*x*_) by^56^ *G* = *ρ*_*x*_ *RTQ* ^–1/3^.

Similarly, we observed decreasing swelling ratios for increasing monomer concentrations (Fig. 3c; 7.5 wt%, *Q* = 41.4 ± 1.3; 10 wt%, *Q* = 33.3 ± 0.6; 12.5 wt%, *Q* = 29.2 ± 0.4). Increased crosslink density inhibits how much a gel is able to swell, thus the inverse relationship between *ρ*_*x*_ and *Q* is expected and observed. The results for the moduli and swelling ratios also were found to be statistically significant (*p* < 0.05), indicating that the material system may be easily tuned to have specific mechanical properties by varying the concentration of monomer present within a gel.

### Hydrogel stability demonstrated for long-term culture

Hydrogel degradation over time often is desirable for cell culture applications to allow cellular processes, such as growth, proliferation, and migration, which can be constrained or hindered by a tightly crosslinked material.^57^ However, nonspecific degradation in aqueous solutions (*e.g.*, hydrolytic cleavage of bonds within functional groups) can limit the degree of user control over materials properties afforded by the addition of enzymatically degradable peptide crosslinks^18^ or photodegradable chemistries,^58^ resulting in unintended or premature hydrogel degradation such that the gel does not remain intact for appropriate time periods during cell culture. For example, Shih and Lin have shown that step growth PEG-tetranorbornene-based thiol–ene hydrogels completely degrade in 2–3 weeks at physiological pH (pH ∼ 7.4), where the norbornene is linked to PEG by an ester bond leading to hydrolytic degradation. Specifically, the degradation rate of these hydrogels was influenced by the peptide crosslinker sequence, where peptides containing hydrophobic or aromatic residues exhibited slower degradation (*e.g.*, CGGGC sequence *k*_hyd_ = 0.049 ± 0.001 day^–1^, CGGLC sequence *k*_hyd_ = 0.036 ± 0.002 day^–1^).^29^

**Fig. 4:**
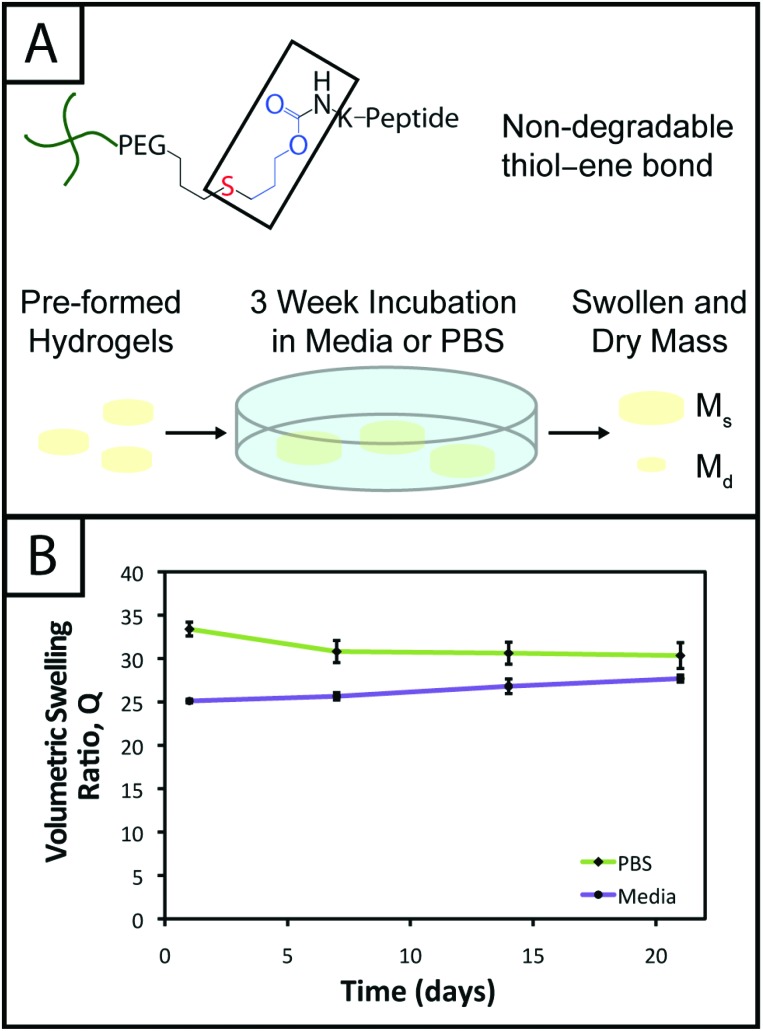
**Hydrogel stability toward long-term cell culture.** (A) The thiol–ene system is linked by a carbamate bond between a thiol-functionalized PEG macromer and an alloc-functionalized peptide, which both lack hydrolytically cleavable bonds (*e.g.*, esters). To evaluate gel stability for controlled cell culture over several weeks, 10 wt% hydrogels (with respect to PEG) were incubated in cell culture medium and PBS for 3 weeks and volumetric swelling ratios (*Q*) measured. (B) The swelling of gels incubated in PBS did not significantly change (*p* > 0.05 for all samples) indicating stability over the time period (*Q* = 33.4 ± 0.8, 30.8 ± 1.3, 30.6 ± 1.3, 30.3 ± 1.5). The swelling of gels incubated in growth media slightly increased through the incubation period such that the first two and last *Q* values are different (*p* < 0.05), although consecutive points are not (*p* > 0.05), which may be attributed partly to non-specific degradation of the hydrogel by enzymes present in the more complex growth medium (*Q* = 25.1 ± 0.2, 25.6 ± 0.4, 26.8 ± 0.8, 27.6 ± 0.4).

In the hydrogel system presented here, we aimed to create monomers free of ester bonds to allow the creation of hydrogels that are stable under cell culture conditions. To assess the stability of the resulting hydrogels, we monitored the volumetric swelling ratio (*Q*) of 10 wt% gels incubated in PBS and cell culture medium at 37 °C over a period of three weeks (Fig. 4), a typical length for many two and three-dimensional cell culture experiments.^3^ For both conditions, the *Q* values qualitatively are constant during the time course, and there is no substantial degradation during the incubation period. Quantitatively, the *p*-values for the gels incubated in PBS for different times are all greater than 0.05, indicating no statistical significance between the gels for each time points and thus that degradation does not occur. For the gels incubated in culture medium, when comparing days 1–14, the *p*-values are all greater than 0.05. However, the day 21 time point is statistically different from the day 1 and 7 time points (*p* < 0.05), indicating a slight change in swelling by 3 weeks. We hypothesize that nonspecific degradation of the peptide crosslinker could be occurring over time in growth medium, which is more complex than PBS and contains serum laden with enzymes, resulting in this small but statistically significant increase in swelling. Despite this small swelling change, the hydrogels remain robust and intact over multiple weeks in culture. The swelling ratios of hydrogels in PBS *versus* media also are statistically significant for the entire incubation period, which we speculate results from differences in the composition of PBS and growth media. With this base system, various degradable peptide crosslinks derived from ECM proteins (*e.g.*, GPQG↓IWGQ or IPVS↓LRSG derived from collagen I) can be incorporated within the gels to allow cell-controlled matrix degradation, where the degradation rate of the matrix can be tuned by peptide selection for different applications.^18^

### Biochemical cues spatially patterned within hydrogels

One benefit that photoclick chemistry provides in the design of hydrogels is the ability to control the presentation of biochemical cues in space or time.^59^ Native tissues are dynamic environments with gradients and defined regions of biological cues occurring at different times, and the ability to capture this complexity in synthetic systems is important for understanding and directing cellular processes.^25^ Here, we studied the photoaddition of a model biochemical cue to our material (i) to establish if excess free thiols could be modified after hydrogel formation and (ii) to demonstrate control over the spatial presentation of these cues. Specifically, an alloc-modified integrin binding peptide (RGDS or AF488RGDS) was coupled homogenously or in specific regions to hydrogels containing free thiols using photopatterning.

**Fig. 5:**
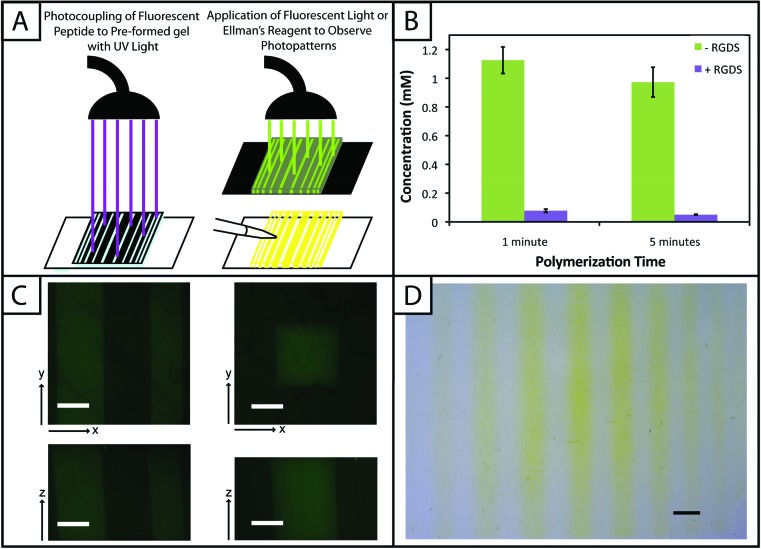
**Biochemical cues spatially patterned within hydrogels.** (A) To create patterns of biochemical cues, hydrogels are polymerized off-stoichiometry ([SH] > [alloc]) and incubated with excess pendant RGDS or AF488RGDS peptide. Gels were irradiated through photomasks printed with black lines or squares for 1 and 5 minutes (left). Samples are subsequently analyzed with fluorescent light or Ellman's reagent to determine the modification of free thiols with pendant biochemical cues (right). (B) Gels (10 wt% with respect to PEG) were polymerized with 2.2 mM LAP for 1 and 5 minutes off stoichiometry (2 mM free thiol at preparation). After equilibrium swelling, the initial free thiol concentration in these gels were 1.13 ± 0.09 and 0.97 ± 0.10 mM, respectively, as determined by Ellman's assay. Only 0.01 ± 0.01 and 0.003 ± 0.003 mM free thiol remained after adding the RGDS tether indicating the efficient coupling of the model biochemical cue to the hydrogel network. (C) Following the setup shown in (A), arbitrary patterns (squares, 1600 μm^2^; lines of different thickness, 200–1000 μm) of a fluorescent peptide (AF488RGDS) were created within pre-formed hydrogels and imaged on a confocal microscope for analysis. Resolution of the pattern is observed in the *x*-, *y*-, and *z*-planes indicating selective coupling to only regions of the gel that were exposed to light (scale bar, 200 μm). (D) As a quick and inexpensive alternative to fluorescence, a non-fluorescent pendant peptide (RGDS) was photopatterned (lines of different thickness) into pre-formed hydrogels. Ellman's reagent was directly applied to the top of these gels to identify regions lacking the pendant peptide (yellow) with resolution in the *x*- and *y*-planes over short times (<5 min) (scale bar, 1 mm).

While one of our goals was to develop a hydrogel from accessible materials, we also aimed to use simple techniques to characterize this system. Ellman's assay, which can identify free thiols in solution, is one such technique that has been used to quantify free thiols in materials post-polymerization.^60,61^ We have utilized this assay in a non-destructive method to quantify free thiols in our hydrogels such that, if desired, gels may be rinsed of reagent, treated with tris(2-carboxyethyl)phosphine (TCEP), rinsed of TCEP, and re-used in additional studies. In addition to quantifying free thiols, we wanted to demonstrate that Ellman's reagent also could be used to observe biochemical patterns created in gels with a reasonable degree of resolution as an inexpensive and rapid alternative or complementary approach to using a fluorescently-tagged cue (Fig. 5a).

Toward achieving this, 10 wt% gels (0.254 mm thick between glass slides) initially were polymerized off stoichiometry so that free thiols (2 mM at preparation prior to equilibrium swelling) remained for later modification with the pendant RGDS peptide. Adjusting for swelling, the free thiol concentration at equilibrium was estimated to be roughly ∼0.61 ± 0.05 mM, so the free thiol concentration in hydrogels as measured by Ellman's assay will be lower than 2 mM. The free thiol concentrations of off-stoichiometry gels polymerized for 1 and 5 minutes subsequently was determined by Ellman's assay to be 1.13 ± 0.09 mM and 0.97 ± 0.10 mM, respectively (Fig. 5b, –RGDS condition). The gels polymerized for 1 and 5 minutes do not have statistically different thiol concentrations (*p* > 0.05), supporting the results in Fig. 2b that the 10 wt% gels are completely formed in under one minute.

To initially determine if a model biochemical cue could be added to these gels, pre-formed gels incubated in RGDS monomer (20 mg mL^–1^ ∼20× excess to SH) with LAP (2.2 mM) were exposed to UV light for 1 and 5 minutes. The thiol concentration after modification was determined for each condition by Ellman's (1 min = 0.01 ± 0.01 mM, 5 min = 0.003 ± 0.003 mM) (Fig. 5b, +RGDS condition). These concentrations correspond to 93.1 and 94.8% modification of the remaining free thiols and 98.5 and 99.0% total thiol modification, indicating high coupling efficiency of the pendant peptide. There are slightly fewer free thiols in the gels polymerized for 5 minutes indicating that a longer polymerization time results in higher conversion of functional groups; however, there is no statistical significance between the two conditions indicating that the effects of longer polymerization are ultimately negligible. Hydrogels polymerized off-stoichiometry (2 mM free thiol at preparation) were then incubated in growth medium at 37 °C for 3 days to determine if cues could be added at different times during culture. Only trace free thiols were observed with Ellman's assay after this 3-day incubation (0.008 ± 0.002 mM), indicating the formation of disulfides either with components in the culture medium or between free thiol end groups on PEG. To test this hypothesis, TCEP (10 mM in PBS) was added to the gels for 1 hour to break potential disulfide bonds. Gels subsequently were rinsed, and the presence of free thiols was detected with Ellman's (1.54 ± 0.09 mM) (ESI Fig. S5†). This recovery of thiols confirms that a large portion of free thiols post-polymerization were lost to disulfide formation upon incubation in culture medium. While the application TCEP could be investigated as an approach to allow temporal photopatterning, reducing agents such as it will negatively affect cell viability^62^ and may not be a practical option for *in situ* photopatterning. However, different orthogonal chemistries^2^ could be utilized within this base hydrogel system to allow the temporal addition of cues throughout long-term cell culture in future investigations.

With the ability to add cues to the matrix after initial formation, spatially defined regions of various cues of interest can be created toward directing the organization and function of cells in three dimensions.^10,22,63^ Fluorescently-labeled cues are typically used to observe biochemical patterns in hydrogel-based matrices with a high degree of resolution; however, this approach requires additional expense and time for peptide labeling and fluorescence imaging. For a rapid and inexpensive assessment of patterning, we examined using Ellman's reagent to observe spatially-defined patterns as a simple alternative or complementary approach for preliminary evaluations. Hydrogels photopatterned with the AF488RGDS peptide demonstrate spatial resolution of cue addition (Fig. 5c) in the *x*, *y*, and *z*-directions for patterns of arbitrary shapes (wide and narrow lines, squares). Next, to test Ellman's as an alternative to fluorescently-labeled evaluation, non-labeled RGDS was patterned into gels, and the gels were imaged under a light microscope immediately after the application of Ellman's reagent (Fig. 5d). At short time periods (<5 minutes), we observed resolution of the patterns; however, as the products from reaction with Ellman's reagent diffused throughout the gel, the pattern began to disappear (ESI Fig. S6†). While Ellman's reagent is limited by the fast diffusion of the reaction products, resulting in the short-term observation of patterns in the *x*–*y* plane only, we envision using this test in initial or follow-up studies of photopatterning in thiol–ene hydrogels because it is easy to use and provides almost instant results. Initially, one could test the ability to pattern a hydrogel before building or purchasing a more expensive fluorescently-tagged peptide. In later experiments, one could quickly confirm that a different peptide or peptide sequence is patterned into the same system without having to build another labeled peptide and use an epi-fluorescent or confocal microscope.

### Encapsulated stem cells remain viable and metabolically active within patterned and non-patterned hydrogels

Hydrogel systems for cell culture or delivery must not only be cytocompatible, but cells also must be able to withstand their polymerization conditions for encapsulation within the matrix. PEG, the primary component of the materials presented here, has been used in a variety of hydrogel systems owing to its bioinert nature, providing a blank slate for the presentation of peptide sequences or whole proteins to elicit specific cellular responses.^25^ Furthermore, cells must be able to withstand multiple doses of UV light and radical initiator for the creation of biochemical patterns within gels to direct cell behavior in three dimensions.

To evaluate the cytocompatibility of the initial polymerization conditions, we encapsulated adult human mesenchymal stem cells, hMSCs, within non-degradable gels (10 wt%, 2.2 mM LAP, 2 mM RGDS, 3000 cells μL^–1^) polymerized for different lengths of time. Specifically, based on our rheometric measurements, hydrogels were polymerized for the minimum amount of time required to completely polymerize 10 and 12.5 wt% samples (1 minute) and in excess of the minimum amount of time to polymerize 7.5 wt% samples (5 minutes). In addition, cell density was kept low to promote primarily cell–matrix interactions and fully understand the limits of cell viability in the system when encapsulating a dilute, single-cell suspension. Cell viability and metabolic activity subsequently were evaluated 1 and 3 days after polymerization to determine polymerization conditions appropriate for the initial encapsulation and culture of cells, respectively.

**Fig. 6:**
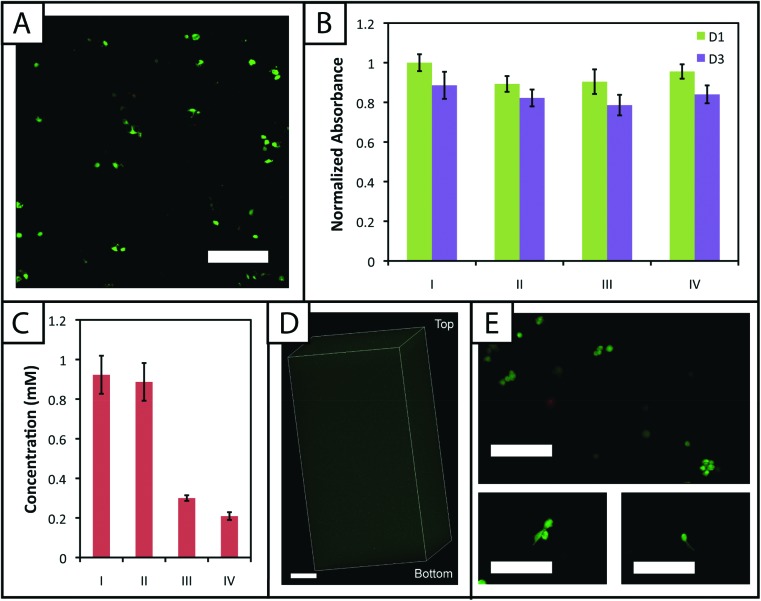
**Encapsulated stem cells remain viable within patterned and non-patterned hydrogels.** (A) Cells were mixed with PEG and peptide monomers and encapsulated to demonstrate the utility of this material system for cell culture in three dimensions. Cells stained green indicate viable cells with intact membranes, whereas cells stained red indicate cells with compromised cell membranes that are dead or dying. Approximately 87 ± 2 and 81 ± 4% of cells were viable after encapsulation and culture for 3 days in 10 wt% gels (with respect to PEG) polymerized for 1 and 5 minutes with 2.2 mM LAP (confocal projection; scale bar, 200 μm). (B) Metabolic activity of the encapsulated cells also was assessed as a second measure of cell viability and function in response to polymerization and short-term culture (normalized to the gels polymerized for 1 minute 1 day after encapsulation). Various encapsulation and photopatterning conditions were tested (I) 1 minute encapsulation, (II) 5 minutes encapsulation, (III) 1 minute encapsulation + 30 minutes PBS/RGDS/LAP + 1 minute photopatterning, (IV) 1 minute encapsulation + 1 hour 30 minutes PBS/RGDS/LAP + 1 minute photopatterning. Condition II for day 1 is statistically different (*p* < 0.05) from I, indicating that longer exposure times to UV and radicals can initially affect viability. However, at day 3, conditions I and II are statistically similar, indicating ‘recovery’ of the cells post-encapsulation. The photopatterning conditions III and IV are statistically similar to I at days 1 and 3; thus, incubation in PBS and a second dose of UV light do not significantly impact cell function. (C) Ellman's assay was conducted on hydrogels processed under conditions used in panel B (I, II, III, and IV). The initial encapsulation conditions I and II have statistically similar free thiol concentrations, consistent with prior gel formation results. The photopatterning conditions III and IV are statistically different from I and II, indicating the ability to covalently attach peptides within the network post-polymerization under mild conditions for cell culture. Further, III and IV are statistically different from each other, suggesting that photopatterning may be diffusion-limited in thicker gels used for cell encapsulation. To evaluate this, (D) gels incubated with 3 mg mL^–1^ AF488RGDS and 2.2 mM LAP for 30 minutes were patterned with a second dose of UV light for 1 minute. Uniform attachment of the fluorescent cue is observed throughout the entire gel depth (∼1.6 mm) (confocal *Z*-stack; scale Bar, 200 μm). (E) Cells encapsulated in a MMP-degradable hydrogel patterned with RGDS remain viable (>90%) over 6 days in culture (top). Several encapsulated cells began to form protrusions by day 6, characteristic of degradation and cell adhesion to the matrix (bottom) (scale bars, 200 μm).

A membrane integrity assay (LIVE/DEAD® Viability/Cytotoxicity Kit) of cells encapsulated in gels (Fig. 6a) showed a higher percentage of living cells in gels polymerized for 1 minute (87 ± 2%) in comparison to 5 minutes (81 ± 4%) at day 3 in culture. While decreased cell viability is observed for the 5 minute polymerization condition, which could limit the use of gels with lower modulus in cell culture (*e.g.*, 7.5 wt%), viability can be rescued by adjustment of experimental parameters, including increased cell–cell contact (*i.e.*, controlling the density of encapsulated cells),^64^ incorporating biomimetic peptides that promote additional cell–matrix interactions,^20,65^ and lower initiator concentration (*i.e.*, reducing concentration of radicals during polymerization but at some cost to polymerization time).^38^ We increased the encapsulation density of cells in non-degradable gels polymerized for 5 minutes (3000 to 30 000 cells μL^–1^) and demonstrated a corresponding increase in viability (83 ± 2% to 92 ± 1%) (ESI Fig. S7†). Accordingly, cell encapsulation density can be adjusted as appropriate to support viability and function depending on the experimental variables to be studied and should be considered in experimental design when using this system.

The metabolic activity of cells, an indicator of cell viability and function, also was monitored 1 and 3 days after encapsulation using CellTiter 96. Constant metabolic activity over time was observed in the gels polymerized for 1 and 5 minutes over three days (*p* > 0.05) (Fig. 6b). Initially (D1) the metabolic activity of the gels polymerized for 5 minutes is statistically different (*p* < 0.05) from gels polymerized for only one minute. However, by day 3, the metabolic activity of the gels polymerized for 1 and 5 minutes is statistically similar (*p* > 0.05), indicating that the initial effects of the polymerization are most apparent for longer irradiation time periods but do not impact cell metabolic activity past the initial treatment. Here, the short-term effects of encapsulation on cell survival appear minimal and similar to that observed in other hydrogels formed by free radical initiation,^38,48^ indicating that this new hydrogel system could support cell culture or delivery in various experimental applications.

Note that all conditions in the metabolic activity experiments presented above were normalized to cells encapsulated in hydrogels with 1 minute of light exposure. While normalization to encapsulated cells without UV exposure is desirable, the hydrogel system presented cannot be easily formed without light. To assess any effect of UV light alone on cell function, hMSCs were seeded in 96-well plates and metabolic activity monitored 1 and 3 days after exposure to UV. Light exposure did not significantly affect hMSC metabolic activity at either D1 or D3 post-irradiation (*p* > 0.05, compared to no UV control) (ESI Fig. S8†). This result is consistent with the reports of others for single doses of UV light at 10 mW cm^–2^.^66^

Toward utilizing this system for patterning gels with biochemical cues during cell culture, we sought to establish relatively mild photopatterning conditions to enable the application of multiple doses of light and radicals within 24 hours of encapsulation. We first incubated gels with 2 mM free thiols prior to swelling in serum-free and serum-containing, phenol red-free growth medium for 2 hours at 37 °C. Only 0.26 ± 0.02 and 0.24 ± 0.04 mM free thiols remained after incubation indicating free thiol consumption at a rate much faster than 24 hours (ESI Fig. S5†); consequently, gels need to be incubated in PBS, rather than culture medium, for photopatterning in the presence of cells. A balance must be struck between allowing time for diffusion of the peptide and initiator into the gels while minimizing the time that cells are incubated in PBS during this process. To address this, we polymerized gels in geometries in which cell encapsulation experiments were conducted (10 wt%, 20 μL gels in syringe tips) for 1 minute and placed them immediately in the patterning solution (PBS containing 3 mg mL^–1^ RGDS ∼3× excess to SH and 2.2 mM LAP). Gels were incubated at 37 °C for 30 minutes or 1 hour and 30 minutes, times longer and shorter than the time estimated for diffusion of the monomer to the center of the gel assuming Fickian diffusion (*t*_d_ ∼ 65 minutes):
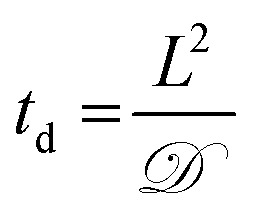
where *L* is half the thickness of the unswollen gel (∼0.625 mm) and 𝒟 the diffusion coefficient (∼10^–6^ cm^2^ s^–1^ based on proteins of similar molecular weight as the RGDS peptide).^67^ A second dose of UV light (1 minute) was applied to covalently link RGDS within the hydrogel. As previously observed, free thiol concentration in gels polymerized for 1 and 5 minutes (without patterning) was not statistically different (*p* > 0.05) and the patterned gels exhibit significantly lower concentrations of free thiols post-patterning (*p* < 0.05 compared to that after 1 and 5 minute gel formation) at 0.30 ± 0.01 and 0.21 ± 0.02 mM, respectively (Fig. 6c). These two photopatterning conditions have statistically different thiol concentrations after polymerization (*p* < 0.05), suggesting that the peptide and initiator may not have fully penetrated the gel during this incubation time. To test this hypothesis, gels (10 wt%, 20 μL in syringe tips, 1 minute polymerization) were incubated with AF488RGDS (3 mg mL^–1^) and LAP (2.2 mM) in PBS for 30 minutes, 1 hour, and 1 hour 30 minutes, and exposed to UV light for 1 minute to allow covalent attachment of the fluorescent peptide. *Z*-Stack images through the entire gel depth (confocal) indicate consistent patterning of the peptide through the gel depth for all conditions (Fig. 6d, ESI Fig. S9†). We speculate that the slight differences seen between the thiol concentrations after patterning by Ellman's assay (Fig. 5b and 6c) are the result of small variations between batches of PEG-4SH monomer and hydrogels or the relative excesses at which the cues were tagged (20× for proof-of-concept and 3× for patterning in the presence of cells).

To compare the effects of these photopatterning conditions on cell activity and viability, cells encapsulated in non-degradable gels (3000 cells μL^–1^, 1 minute UV exposure) were incubated for 30 minutes or 1 hour 30 minutes in PBS containing RGDS and LAP and a second dose of UV light subsequently was applied for 1 minute. Cell metabolic activity for these photopatterning conditions is statistically similar to the 1 minute hydrogel formation condition at days 1 and 3 (*p* > 0.05), indicating that exposure to multiple polymerizations (formation + patterning) has a minimal effect on cell function (Fig. 6b). There appears to be a slight, but not statistically significant, decrease in metabolic activity for each condition between days 1 and 3. We hypothesize that this negligible decrease results from minor damage to cells in all cases by the radically-mediated polymerizations, which shows up in reduced metabolic activity at day 3. No statistical difference is observed between any condition at day 3. Taken together, no specific effect of the photopatterning process is observed, and the photopatterning conditions assessed here are appropriate for use in cell culture.

Finally, toward long-term culture of cells in patterned gels, hMSCs were encapsulated in cell-degradable gels crosslinked with a MMP-cleavable peptide sequence^18^ (GPQGIWGQ2alloc) and treated with 3 mg mL^–1^ RGDS and 2.2 mM LAP in PBS for 1 hour (between the minimum and maximum incubation times tested for photopatterning) before a second dose of UV light was applied to photopattern RGDS within the network. After 6 days of culture, cells were stained with the LIVE/DEAD® Viability/Cytotoxicity Kit and imaged on a confocal microscope to observe cell viability and any spreading within the network. Viability greater than 90% was observed and a few cells exhibited protrusions (Fig. 6e), indicative of adhesion to and degradation of the matrix. Based on these results, this approach for cell encapsulation and matrix photopatterning is promising for future studies to probe stem cell–material interactions and direct cell function and fate *in vitro*.

## Conclusions

In summary, we presented a novel hydrogel system formed by thiol–ene photoclick chemistry through reaction of thiol-modified PEG and alloc-modified peptides. Use of the LAP photoinitiator allowed rapid polymerization with cytocompatible doses of UV light and the formation of hydrogels with appropriate mechanical properties to mimic soft tissues. These hydrogels remain stable in cell culture conditions and encapsulated cells are viable within the network. Biochemical cues were selectively patterned within the gels to demonstrate spatial control over matrix properties, and cells remained viable. Further, the monomers used in the design of this system may be synthesized using established protocols or commercially purchased, making the material accessible for the facile and consistent formation of robust hydrogels to mimic the ECM. In the future, this base material may be used with orthogonal click chemistries to allow control over biochemical and biomechanical properties over days to weeks to study cell response to changes in the surrounding environment and provides a useful platform to adapt for a variety of biomaterials applications, including cell culture, tissue engineering, and drug delivery. Specifically, toward application in culture and directing hMSC fate, gels could be patterned with individual or multiple biochemical cues in spatially defined regions to drive cellular processes, including adhesion, migration, proliferation, or differentiation.^68,69^
