# Supplementary material for: Design of thiol–ene photoclick hydrogels using facile techniques for cell culture applications
Source: Biomater Sci. 2014 Sep 1;2(11):1612–26. doi: 10.1039/c4bm00187g (PMC4324132; doi:10.1039/c4bm00187g)
Supplement: Supplementary file 1 [file BM-002-C4BM00187G-s001.pdf]

Electronic Supplementary Material (ESI) for Biomaterials Science.
This journal is © The Royal Society of Chemistry 2014

Design of Thiol–ene Photoclick Hydrogels Using Facile Techniques for Cell Culture Applications

Lisa A. Sawicki,[a] *April M. Kloxin*[a,b]

[a]Department of Chemical and Biomolecular Engineering, University of Delaware, Newark, DE 19716, USA. Email: akloxin@udel.edu

[b]Department of Materials Science and Engineering, University of Delaware, Newark, DE 19716, USA.

SUPPLEMENTARY INFORMATION

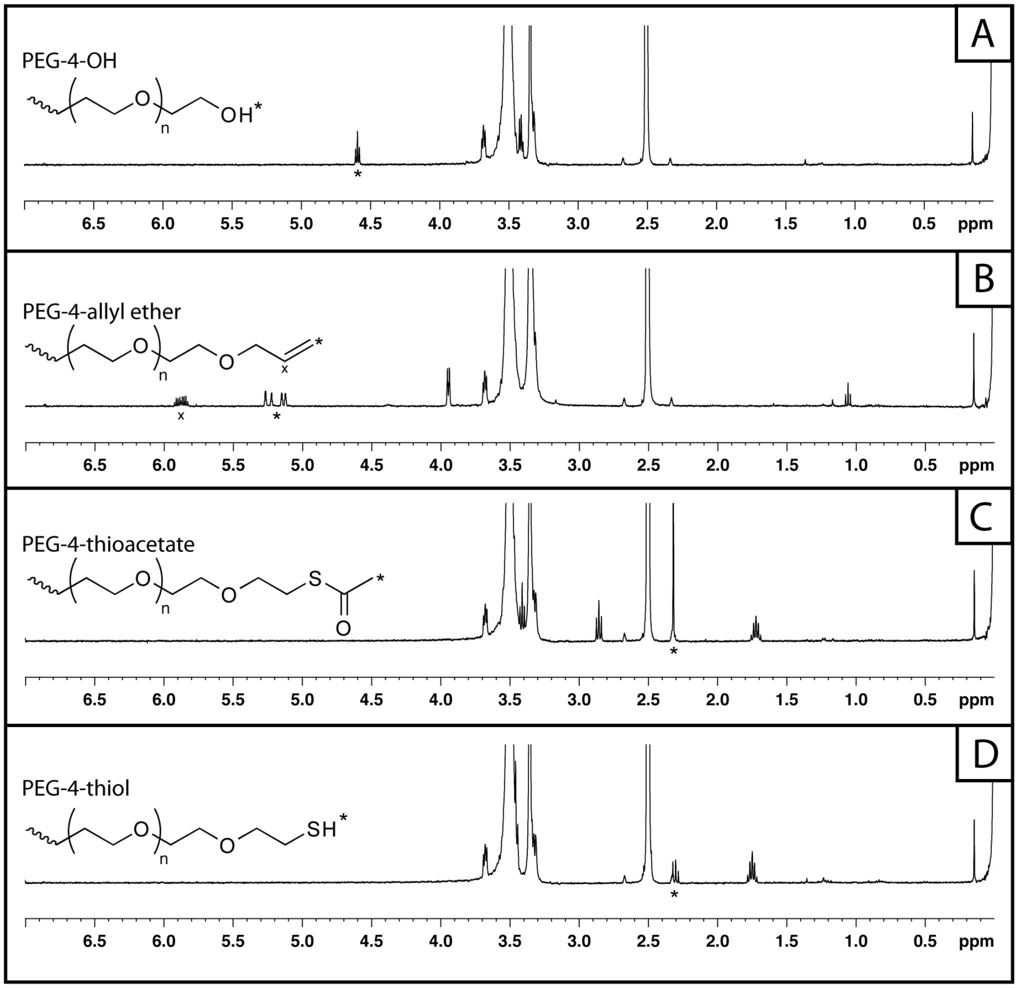

Supplemental Figure S1. ^{1}H NMR of product and intermediates in PEG4SH synthesis in DMSO. **A)** The hydrogen on the hydroxyl group of the initial PEG 20k monomer is identified at 4.6 (m, 1H). **B)** After reaction with allyl bromide, the hydroxyl peak on the PEG disappears and allyl ether peaks appear at 5.1-5.2 (m, 1H), 5.2-5.3 (m, 1H), and 5.8-5.9 (m, 1H), respectively. **C)** Conversion of the PEG-4-allyl ether (PEG4AE) to PEG-4-thioacetate results in

the disappearance of allyl ether associated peaks, replaced by a peak at 2.3 (s, 3H), corresponding to the methyl group on the thioacetate. **D)** After final conversion of PEG-4-thioacetate, the product PEG-4-thiol (PEG4SH) was confirmed by the presence of a peak at 2.3 (m, 1H) indicating the hydrogen on free thiol groups. This peak has the same shift as that of the thioacetate peak, but is a multiplet and corresponds to one hydrogen.

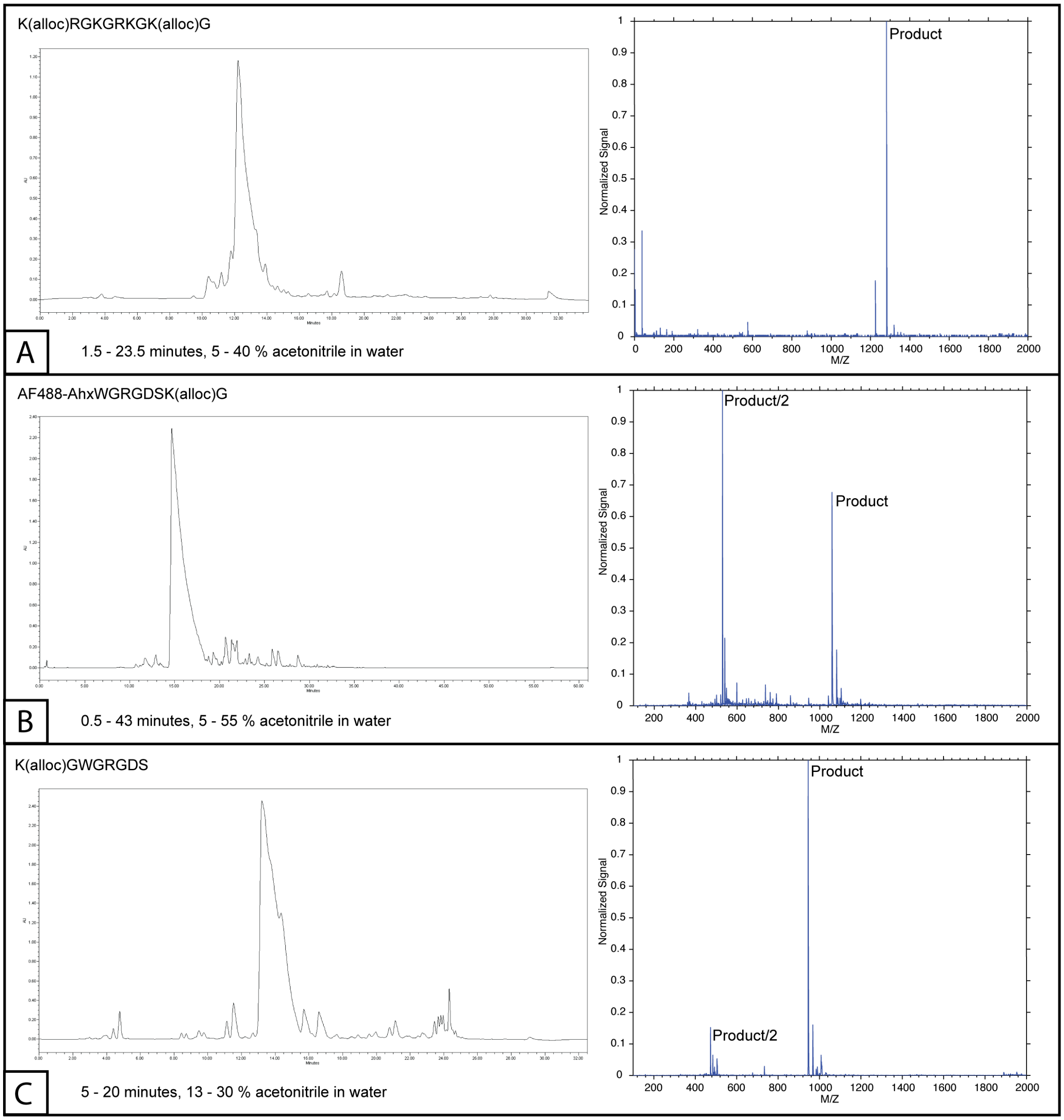
K(alloc)RGKGRKGK(alloc)G
Product
Normalized Signal
M/Z
A
1.5 - 23.5 minutes, 5 - 40 % acetonitrile in water
AF488-AhxWGRGDSK(alloc)G
Product/2
Product
Normalized Signal
M/Z
B
0.5 - 43 minutes, 5 - 55 % acetonitrile in water
K(alloc)GWGRGDS
Product
Product/2
Normalized Signal
M/Z
C
5 - 20 minutes, 13 - 30 % acetonitrile in water

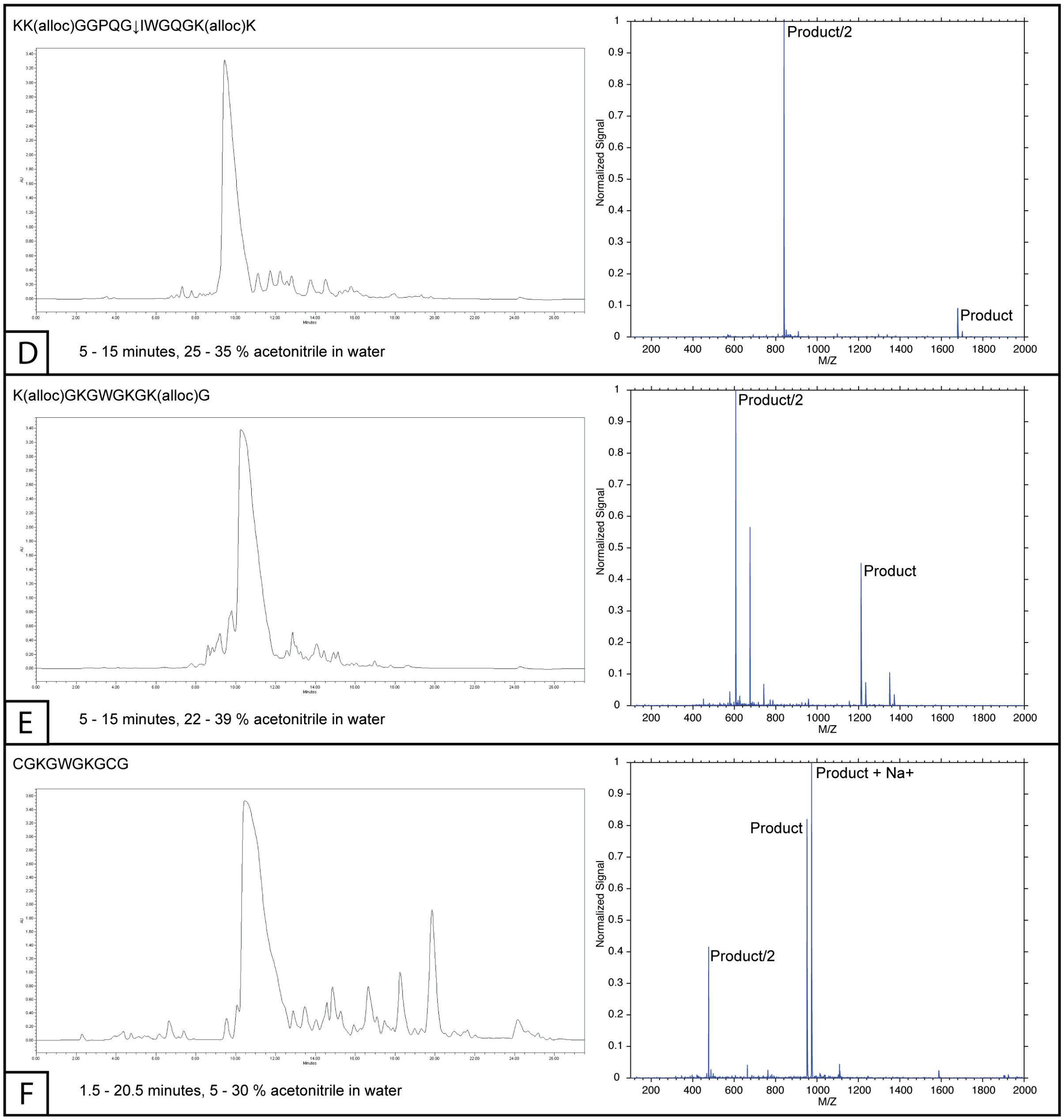

Supplemental Figure S2. HPLC traces, MALDI-MS, and ESI-MS spectra for the purification and identification of synthesized peptides. A) The major peak observed with HPLC (left) was collected for the purification of K(alloc)RGKGRKGK(alloc)G (RGKGRK2Alloc) crosslinker peptide. The final product was confirmed by MALDI-MS (MW = 1282 g/mol, N'-terminus acetylation). **B)** The major peak observed with HPLC (left) contains AF488-AhxWGRGDSK(alloc)G (AF488RGDS). The crude product was analyzed by ESI-MS (MW = 1059 g/mol). Note that the molecular weight is for AhxWGRGDSK(alloc)G because the AF488AhxWGRGDSK(alloc)G is a minor species (approximately 1 for every 220 AhxWGRGDSK(alloc)G). **C)** The major HPLC peak (left) for the pendant K(alloc)GWGRGDS (RGDS) was analyzed by ESI-MS and the final product confirmed. (MW = 946 g/mol). **D)** The

major HPLC peak (left) analyzed by ESI-MS (right) confirmed the synthesis of KK(alloc)GGPQG↓IWGQGK(alloc)G (GPQGIWGQ2alloc, MW = 1680 g/mol, N'-terminus acetylation). **E)** The major HPLC peak (left) of K(alloc)GKGWGKGK(alloc) (GKGWGKG2alloc) was analyzed by ESI-MS to confirm synthesis of the final product (MW = 1213 g/mol, N'-terminus acetylation). **F)** The HPLC peak (left) of CGKGWGKGCG (GKGWGKG2SH) was analyzed by ESI-MS and the final product confirmed (MW = 952 g/mol).

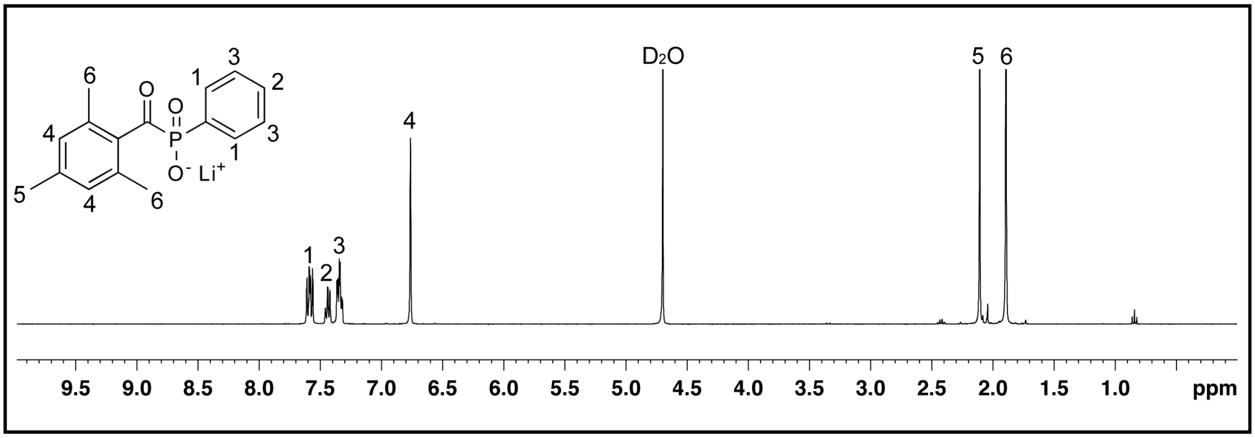

Supplemental Figure S3. ^{1}H NMR of lithium acylphosphinate initiator in D_2O. Peaks labeled 1-6 are identified on the NMR spectra corresponding to hydrogens on each ring of the initiator.

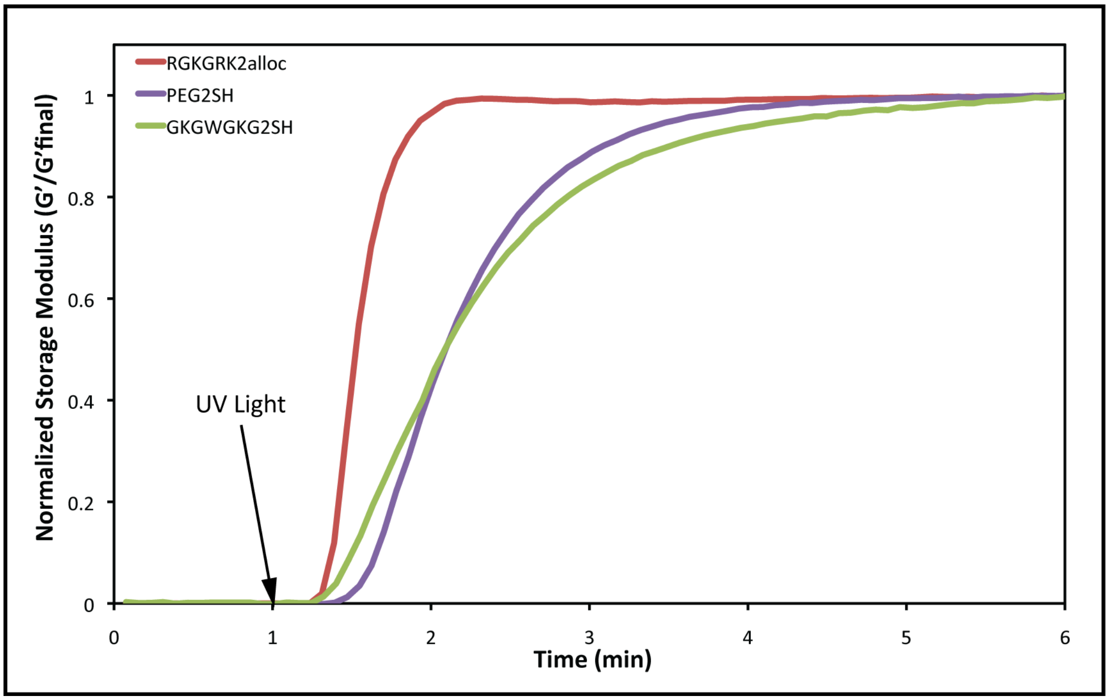

Supplemental Figure S4. Polymerization of thiol–ene hydrogels with various peptides and functionalities. Hydrogels (10 wt%) were polymerized with different initial monomer solutions to investigate the effects of peptide sequence and functionality on polymerization rate. The highly charged RGKGRK2alloc crosslinking peptide exhibits the fastest polymerization when reacted with PEG4SH. The PEG2SH crosslinking molecule, when reacted with PEG4AE, exhibits a slower polymerization than RGKGRK2alloc with PEG4SH, which may be due to either the lack of charge in the reacting monomers or the change of the reacting functional groups. Finally, the GKGWGKG2SH peptide with PEG4AE exhibited the slowest reaction, with the modulus continuing to slowly increase at 6 minutes. This reaction may be partly affected by functional group chemistry as well as disulfides, as the moduli (normalized here to the final modulus at 6 minutes) were an order of magnitude lower than the other gel conditions tested and the modulus appeared to continue to increase slowly over time. Ellman's assay was used to measure free thiols in the peptide stock solution to insure 1:1 stoichiometry and total peptide concentration also was checked with absorbance at 280 nm; however, this decreased polymerization rate and modulus were consistently observed.

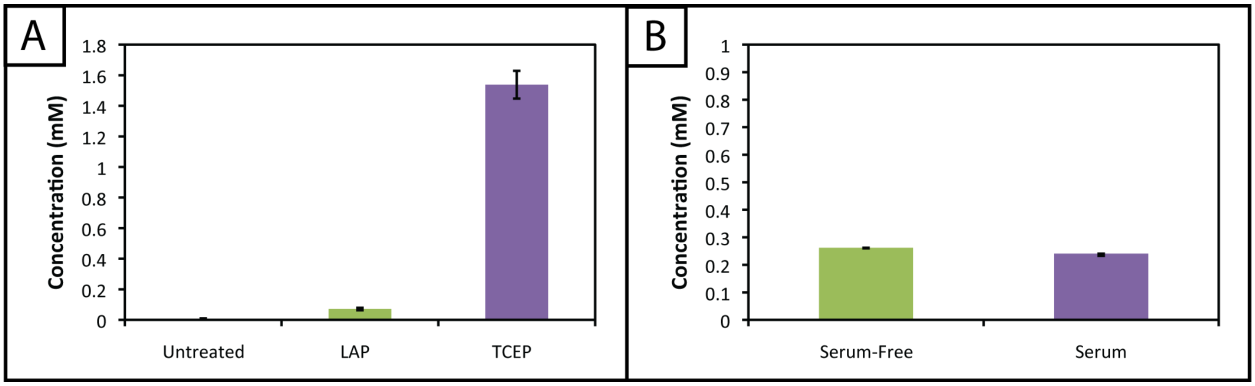

Supplemental Figure S5. Ellman's assay to determine free thiol concentration in hydrogels over time in cell culture medium. A) Hydrogels incubated in growth medium for 3 days were analyzed by Ellman's assay to determine if culture conditions affected temporal addition of biochemical cues. After 3 days in culture medium, only trace thiols were detected in the 'Untreated' condition by Ellman's assay (< 0.01 mM), indicating the formation of disulfide bonds over time. To test the hypothesis that disulfides were forming, TCEP, a strong reducing agent, was added to the samples and free thiol concentrations were found to be 1.54 ± 0.09 mM, indicating that extensive disulfide formation occurred during incubation with cell culture medium. In the literature, LAP has been shown to cleave disulfide bonds in disulfide-bonded PEG-tetrathiol hydrogels, and we wanted to determine if this could be applied in a PEG-peptide system.[43] Samples were incubated with LAP and irradiated with UV light. A slightly higher concentration of free thiols was present after irradiation with light (0.07 ± 0.01 mM); however, this concentration would not be a sufficient for the addition of biochemical cues to direct cell function. In future studies, orthogonal click chemistries will be investigated to allow long-term temporal control over biochemical properties. **B)** Hydrogels incubated in serum-free and serum-containing phenol red-free cell culture medium for 2 hours have decreased thiol concentrations (0.26 ± 0.02 and 0.24 ± 0.04 mM), indicating rapid consumption of free thiols in culture medium and that photopatterning must be performed immediately in PBS.

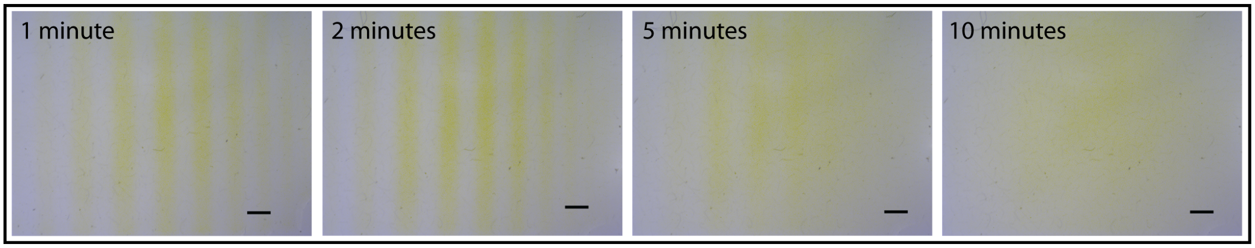

Supplemental Figure S6. Discrete time lapse of photopatterned hydrogels in the presence of Ellman's reagent. A photopatterned hydrogel was incubated with Ellman's reagent, and imaged at 1, 2, 5, and 10 minutes to monitor diffusion of the yellow TNB^{2-} ion through the sample. At 1 and 2 minutes, unpatterned regions are vibrant yellow; however, as time increases to 5 minutes, the pattern becomes less resolved and eventually disappears by 10 minutes. Samples should be imaged immediately for the best resolution of patterns. (Scale bars, 200 μm)

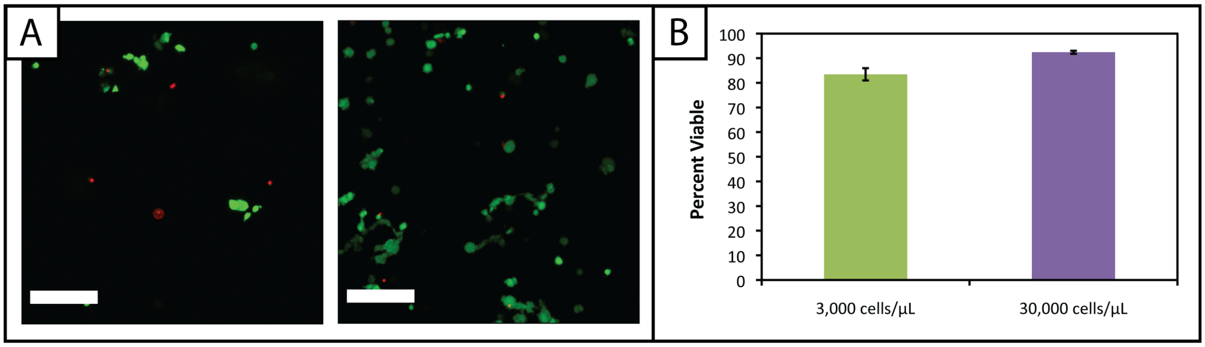

Supplemental Figure S7. Cell encapsulation and viability at different seeding densities. **A)** Viability of cells encapsulated in 10 wt% hydrogels at 3000 (left) and 30000 (right) cells/µL was determined 3 days after encapsulation. Live (green) and dead (red) cells were identified with a LIVE/DEAD® Viability/Cytotoxicity Kit. (Scale bars, 200 µm) **B)** Approximately 83 ± 2% of cells remain viable in gels with 3000 cells/µL, whereas a statistically higher percent of cells ($p < 0.05$) remain viable in gels with 30000 cels/µL at 92 ± 1%.

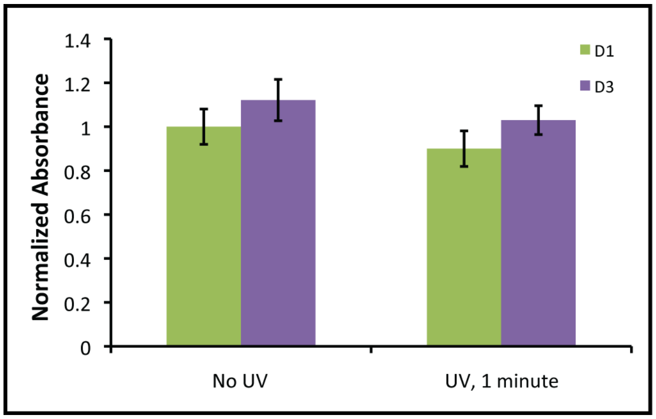

Supplemental Figure S8. Metabolic activity of cells treated with UV light in 2D. Cells seeded on plates were treated with UV light for 1 minute. Metabolic activity was monitored 1 and 3 days after treatment (D1 and D3) to determine the potential effects. All absorbances were normalized to the "No UV" condition at D1. Exposure to UV light alone does not have an apparent effect on metabolic activity ($p > 0.05$) when compared to the "No UV" condition. Further, an increase in metabolic activity is seen at D3, indicating that the cells are able to recover from (and potentially proliferate after) exposure.

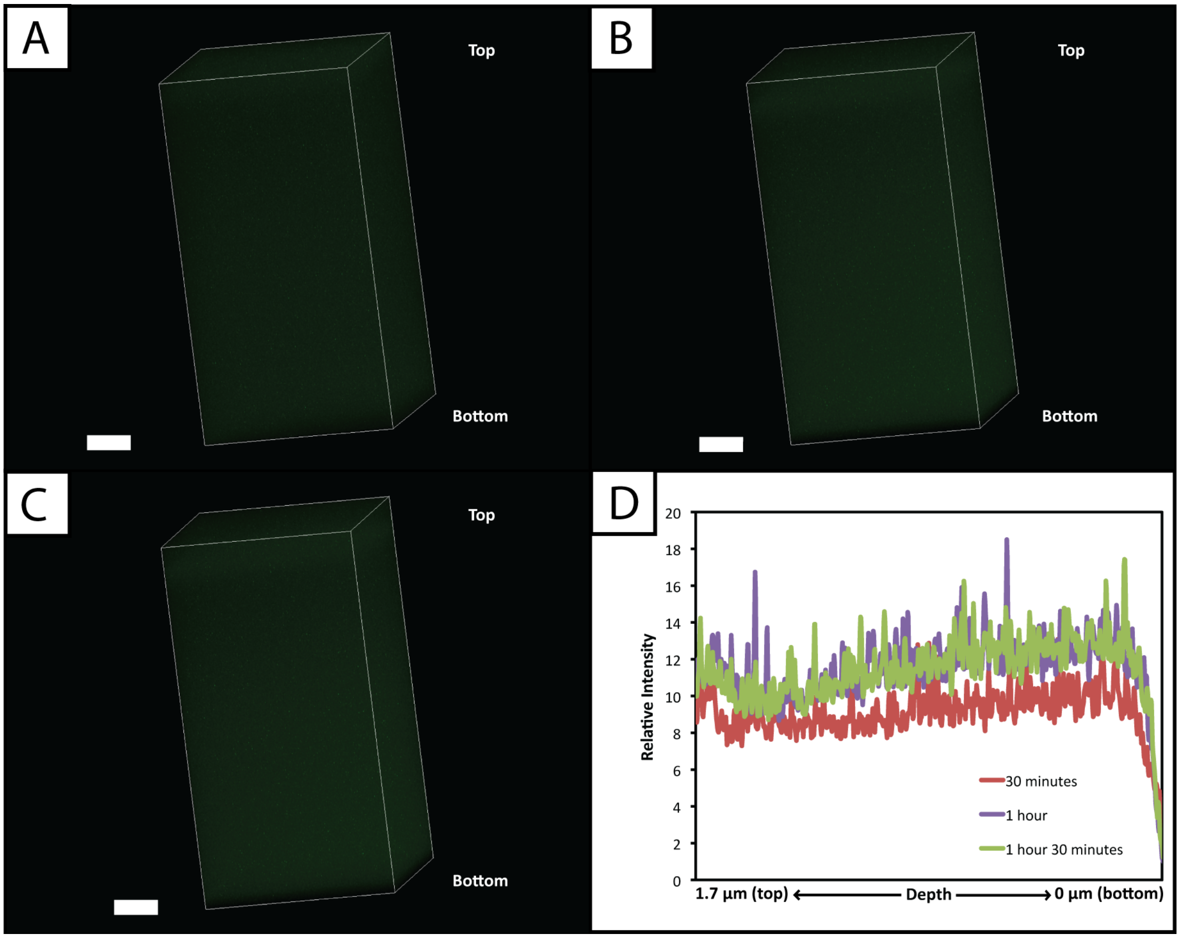

Supplemental Figure S9. Whole gels patterned with AF488RGDS to determine depth of diffusion and patterning. Gels (20 µL) were incubated with photopatterning solution (3 mg/mL AF488RGDS, 2.2 mM LAP, PBS) for **A)** 30 minutes, **B)** 1 hour, and **C)** 1 hour 30 minutes, before applying a second dose of UV light for 1 minute. Confocal imaging through the gel depth indicates attachment and uniform patterning of the fluorescent peptide. (Scale bars, 200 µm) **D)** The relative intensity of the fluorophore for all three conditions is approximately the same (within noise), indicating that the incubation times are sufficient to achieve uniform patterning throughout the gel. The decrease in intensity at 0 µm indicates the bottom of the gel has been reached (also seen in A-C, where the image is darker).
